# Computational assessment of the functional role of sinoatrial node exit pathways in the human heart

**DOI:** 10.1371/journal.pone.0183727

**Published:** 2017-09-05

**Authors:** Sanjay R. Kharche, Edward Vigmond, Igor R. Efimov, Halina Dobrzynski

**Affiliations:** 1 Institute of Cardiovascular Sciences, School of Medical Sciences, University of Manchester, Manchester, United Kingdom; 2 University of Bordeaux, IMB, UMR 5251, Talence, France; 3 IHU Liryc, Electrophysiology and Heart Modeling Institute, Fondation Bordeaux Université, Pessac- Bordeaux, France; 4 Department of Biomedical Engineering, The George Washington University, Washington, DC, United States of America; Universiteit Gent, BELGIUM

## Abstract

**Aim:**

The human right atrium and sinoatrial node (SAN) anatomy is complex. Optical mapping experiments suggest that the SAN is functionally insulated from atrial tissue except at discrete SAN-atrial electrical junctions called SAN exit pathways, SEPs. Additionally, histological imaging suggests the presence of a secondary pacemaker close to the SAN. We hypothesise that *a) an insulating border-SEP anatomical configuration is related to SAN arrhythmia; and b) a secondary pacemaker*, *the paranodal area*, *is an alternate pacemaker but accentuates tachycardia*. A 3D electro-anatomical computational model was used to test these hypotheses.

**Methods:**

A detailed 3D human SAN electro-anatomical mathematical model was developed based on our previous anatomical reconstruction. Electrical activity was simulated using tissue specific variants of the Fenton-Karma action potential equations. Simulation experiments were designed to deploy this complex electro-anatomical system to assess the roles of border-SEPs and paranodal area by mimicking experimentally observed SAN arrhythmia. Robust and accurate numerical algorithms were implemented for solving the mono domain reaction-diffusion equation implicitly, calculating 3D filament traces, and computing dominant frequency among other quantitative measurements.

**Results:**

A centre to periphery gradient of increasing diffusion was sufficient to permit initiation of pacemaking at the centre of the 3D SAN. Re-entry within the SAN, micro re-entry, was possible by imposing significant SAN fibrosis in the presence of the insulating border. SEPs promoted the micro re-entry to generate more complex SAN-atrial tachycardia. Simulation of macro re-entry, i.e. re-entry around the SAN, was possible by inclusion of atrial fibrosis in the presence of the insulating border. The border shielded the SAN from atrial tachycardia. However, SAN micro-structure intercellular gap junctional coupling and the paranodal area contributed to prolonged atrial fibrillation. Finally, the micro-structure was found to be sufficient to explain shifts of leading pacemaker site location.

**Conclusions:**

The simulations establish a relationship between anatomy and SAN electrical function. Microstructure, in the form of intercellular gap junction coupling, was found to regulate SAN function and arrhythmia.

## Introduction

The cardiac impulse generated in the sinoatrial node (SAN) propagates to the atrium through SAN-atrial junctions and to the rest of the heart through the cardiac conduction system [1]. Whereas sinus tachy-brady arrhythmia are identified clinical concerns, their mechanistic understanding remains restricted for a spectrum of reasons including a limited understanding of the cardiac anatomy in the SAN region of the heart [2].

Modern computational cardiology is used to quantitatively unveil pathophysiological arrhythmia mechanisms and explore therapies [3]. In contrast to sophisticated human 3D ventricular models, the current generation of SAN models focus on electrophysiological disorders relying on simple SAN anatomies [4, 5]. To address the limitation, two dimensional models of SAN capable of assessing existing conjectures as developed by the Vigmond group [6] are less prevalent. However, some two dimensional models are simplified representations of thin transmural SAN electro-anatomy [7], a feature that limits their applicability. The electrically heterogeneous SAN has been implemented in the 3D human atrial model by Seemann et al. [8], but the experimental evidence for SEPs was yet to be generated. More recently, Li et al. [9] developed a detailed 3D anatomical model of the rabbit atrium using high resolution (~ 24 μm) DT-MRI imaging. In their study, Li et al. studied the interaction between the SAN and atrioventricular node. It can be appreciated that the role of SEPs would be secondary to the other heterogeneities that Li et al. have studied. Apart from SEPs, experimental evidence [10] as well as theoretical studies [11, 12] have shown the relevance of atrial strands interdigitating into SAN tissue. Whereas SAN interdigitations are a relevant SAN anatomical feature, this study focus’ on the role of SEPs in SAN function within the immediate anatomical vicinity of the human SAN.

Arrhythmia is a manifestation of the complex electrical propagations dictated by anatomy, intercellular gap junction coupling microstructure, and electrophysiology under pathological conditions. Detailed 3D human cardiac anatomy the SAN region has been quantified using histological methods in the Dobrzynski group [9, 13, 14]. The histological evidence strongly suggests the presence of a secondary pacemaker in close vicinity of the SAN [14]. However, the SAN may be electrically coupled to the surrounding atrium at only a few discrete locations called SAN exit pathways (SEPs), something that has eluded conventional imaging of the anatomy. The application of optical mapping methods to study right atrial electrical propagations developed in the Efimov group [15] was crucial in providing evidence for the existence of SEPs. A number of SAN related electrical propagation patterns can be expected to be affected by SEPs.

Functional experiments in right atrial preparations have suggested that propagating electrical waves are channelled in and out of the SAN at specific locations, i.e. through the SEPs [16]. Under certain conditions “macro re-entry” has been observed when electrical waves become bound to the SAN’s exterior giving rise to atrial flutter or tachycardia [17]. The biophysical factors that permit macro-re-entry remain under investigated.

Re-entry within the SAN has been observed in post myocardial infarction dogs by Glukov et al. [18], which may also occur in the human SAN. In their study, Glukhov et al. observed a persistent re-entry under the action of isoprenaline. Such intra-SAN re-entry has been termed as “micro re-entry” which was attributed to intra-nodal fibrosis. However, elucidating the nature of the fibrosis [19] may not be feasible experimentally due to the small size of the SAN and inter-individual variability. Prolonged episodes of such “micro re-entry” have been observed when circulating waves persistently exist within the SAN, and give rise to complex SAN-atrial propagations [17, 18, 20]. A slow conduction velocity of experimentally observed values between 3 and 12 cm/s within the SAN may contribute to stability of micro re-entry [20]. Although the role of border-SEPs in sustained micro re-entry is becoming clear using experimental methods, the contribution of important factors such as intercellular coupling dysfunction remain to be explored.

Application of pharmacological agents is known to induce a shift in the leading pacemaker site (LPS) [21, 22] and may be affected by SEPs. Another consideration is that an insulating border-SEP configuration can be expected to shield the SAN from atrial tachycardia. These events in the proximity of the SAN are likely to be affected by the anatomy. The presence of a secondary pacemaker may also affect the events within the SAN, as well as participate in physiopathological pacemaking itself. However, additional electro-microstructural alterations may also be a necessary mechanistic component underlying the occurrence of these phenomena, a crucial factor that remains unclear to date.

In this computational study, the anatomical SEPs and micro-structural cell-cell coupling representing intercellular gap junctions are linked to micro re-entry, macro re-entry, shift of leading pacemaker site, and degeneration of tachycardia into fibrillation. Although the literature reflects the complex nature of cell-cell coupling heterogeneity or fibrosis [23], this study aimed at reproducing the observed phenomena using relatively simple constructs and a single re-entry. The experimental data [18, 20] also show the effect of acetylcholine and isoprenaline, both biochemicals secreted by nerve endings within the SAN, on shifting the leading pacemaker site within the SAN. Multiple modelling studies illustrate the electrophysiological basis for the leading pacemaker site (LPS) shift [4, 6]. However, cardiac tissue micro-structure in terms of cell-cell coupling may also be locally affected by similar acting biochemicals [24] as shown in this study.

In this study, a *de novo* 3D electro-anatomical model was constructed and used to examine SAN electro-anatomy in light of recent experimental findings. The electrophysiology was modelled using simple cell models and therefore may be considered to be phenomenological. The main aim of this study was to ascertain if anatomical SEPs can be related to arrhythmias observed in the vicinity of the SAN. Specifically, our goals were to:

1. Implement a functional electro-anatomical model of the human SAN;
2. Identify representative fibrosis conditions that permitted persistent micro re-entry and macro re-entry;
3. Demonstrate the functional role of the paranodal area; and
4. Demonstrate the shift of leading pacemaker shift due to altered SAN micro-structure.

## Methods

### 2.1 Model construction

**Fig 1:**
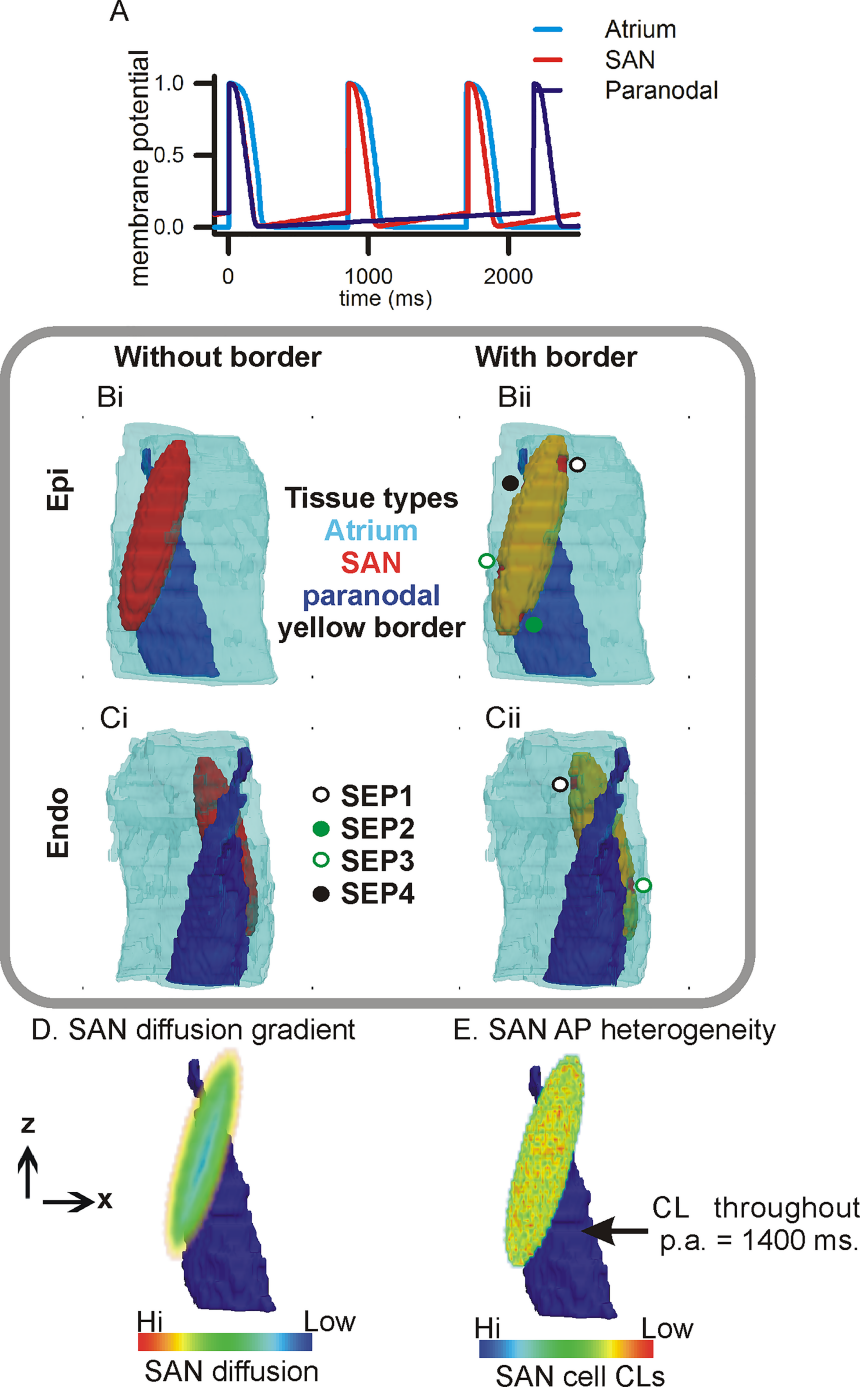
3D electro-anatomical human SAN model. A: Action potential profiles of atrial (cyan), SAN (red), and paranodal area (blue) cells. Gray box: Epicardial (Bi and Bii) and endocardial (Ci and Cii) views of the 3D anatomy consisting of atrial tissue (cyan), SAN (red), paranodal area (blue), insulating border (yellow tissue encasing of the SAN in Bii and Cii), and SEPs. Left panels (Bi, Ci) show the anatomy without border-SEPs. Right column (Bii, Cii) shows the anatomy with the insulating border-SEPs. D: Cell-cell coupling centre to periphery diffusion gradient inside the SAN. The diffusion increases from the SAN’s centroid towards the atrium. The paranodal area (blue) is shown for anatomical reference. E: An instance of uniform distribution of SAN action potential cycle lengths in a range of 800 ms to 1000 ms with a mean of 850 ms. An instance of SAN cell cycle lengths is illustrated in **S1 Fig.** The paranodal area’s cycle length was kept at 1400 ms throughout.

The 3D human SAN electro-anatomical model (3D model) is illustrated in Fig 1.

#### 2.1.1 Baseline electrophysiology

Electrically active cell types were implemented using variants of the Fenton-Karma model [25] for human cell types (Fig 1A). Cell model equations and parameter values are given in the **S1 Section and S1 Table** respectively. The atrial cell type parameters were adopted from a recent study [26] as well as the original model [25] as follows. The parameter regulating the upstroke current (*J*_*fi*_) activation, *u*_*c*_ was set to 0.3. The parameter regulating the upstroke velocity (*τ*_*d*_) was set to 0.2 to permit atrial propagations in our model. The remaining parameters for the atrial cell type were taken from Podziemski and Zebrowski [26]. To simulate a short action potential, time constants in the slow outward and inward current were modified (see **S1 Table**). To simulate the pacemaker cell types of the SAN and paranodal area, the *J*_*fi*_ current and slow inward current, *J*_*si*_, were modified. The parameter *u*_*c*_ was set to 0.1, and (*τ*_*d*_) was adjusted to 0.05 which permitted the SAN cells or paranodal area cells to activate adjacent atrial cells. In the *J*_*si*_ equations, the parameter *τ*_*w*_^*+*^ was set to 1.5, and the cycle length of the two cell types was then regulated by *τ*_*w*_^*-*^. Further, *u*_*c*_^*si*^ was reduced to 0.01. An electrically inactive fourth cell type was included in the model to represent both the insulating border and fibrosis. For simplicity, the inactive cell type was neither considered as a source nor sink, and its function was to stop propagating electrical waves.

It is known that heterogeneous coupled oscillators robustly synchronise as well as sustain function even after deterioration of some individual oscillators, see e.g. [27]. A uniform random distribution of action potentials centred around 850 ms was used to assign to individual SAN cell cycle lengths, which gave a SAN tissue waves at a cycle length of 850 ms [1, 26]. This was achieved by perturbing the parameter τ_w_^-^ (**S1 Table**) randomly from 700 to 900. The range of SAN pacing cycle lengths that this parameter distribution gave was in the range of 750 ms to 945 ms. It also permitted a robust localisation of the LPS site. The paranodal area [14] is expected to be overdrive suppressed during physiological SAN pacemaking. It was therefore arbitrarily assigned a cycle length of 1400 ms, i.e. much longer than that of the SAN. The control atrial action potential duration was set to the experimentally observed value of approximately 260 ms [28, 29]. The ranges of SAN action potentials present in the spatially extended SAN, as well as the atrial action potentials are shown in **S1 Fig.**

#### 2.1.2 Adapted anatomy and anatomical variants

The model anatomy (Fig 1Bi to 1Cii) was adapted from our previous histological-immunohistochemistry study [14]. It consists of a uniform structured grid of 128 x 128 x 60 points. The resolution of the anatomy is 0.25 mm (x direction) x 0.25 mm (y direction) x 0.50 mm (z direction). The original paranodal area and atrial region were incorporated unaltered into the model anatomy. The original SAN was smoothed to an ellipsoidal shape (Fig 1Bi and 1Ci) which permitted: a) the implementation of the insulating border-SEP configuration (Fig 1Bii and 1Cii); and b) smooth diffusion gradient within the SAN as shown in Fig 1D [4, 6] (see below). The original and modified SAN anatomies are illustrated in S2 Fig. The insulating border was assumed to be a 1 voxel thick layer of connective tissue on the SAN surface. The insulating border was perforated at four locations to generate SAN-atria electrical junctions, representing the four SEPs identified in the experimental studies (Fig 1Bii and 1Cii) [15, 20]. To permit comparison, each simulation experiment of electrical events was performed on five anatomical variants, each of which omitted certain components of the full model. In each of the following variants when an anatomical region was omitted, it was replaced by atrial tissue type. The variants considered were:

1. SAN only: The paranodal area and border-SEP were omitted.
2. SAN with border-SEPs: The paranodal area was omitted.
3. SAN with paranodal area: This border-SEPs were omitted.
4. Paranodal only: The SAN, border, and SEPs were omitted.
5. Complete model: This configuration included all anatomical components: a SAN surrounded by border-SEPs, as well as the paranodal area column between the SAN and endocardial atrial wall (Fig 1Bii and 1Cii).

#### 2.1.3 Intercellular gap junction coupling micro-structure modelling

**Fig 2:**
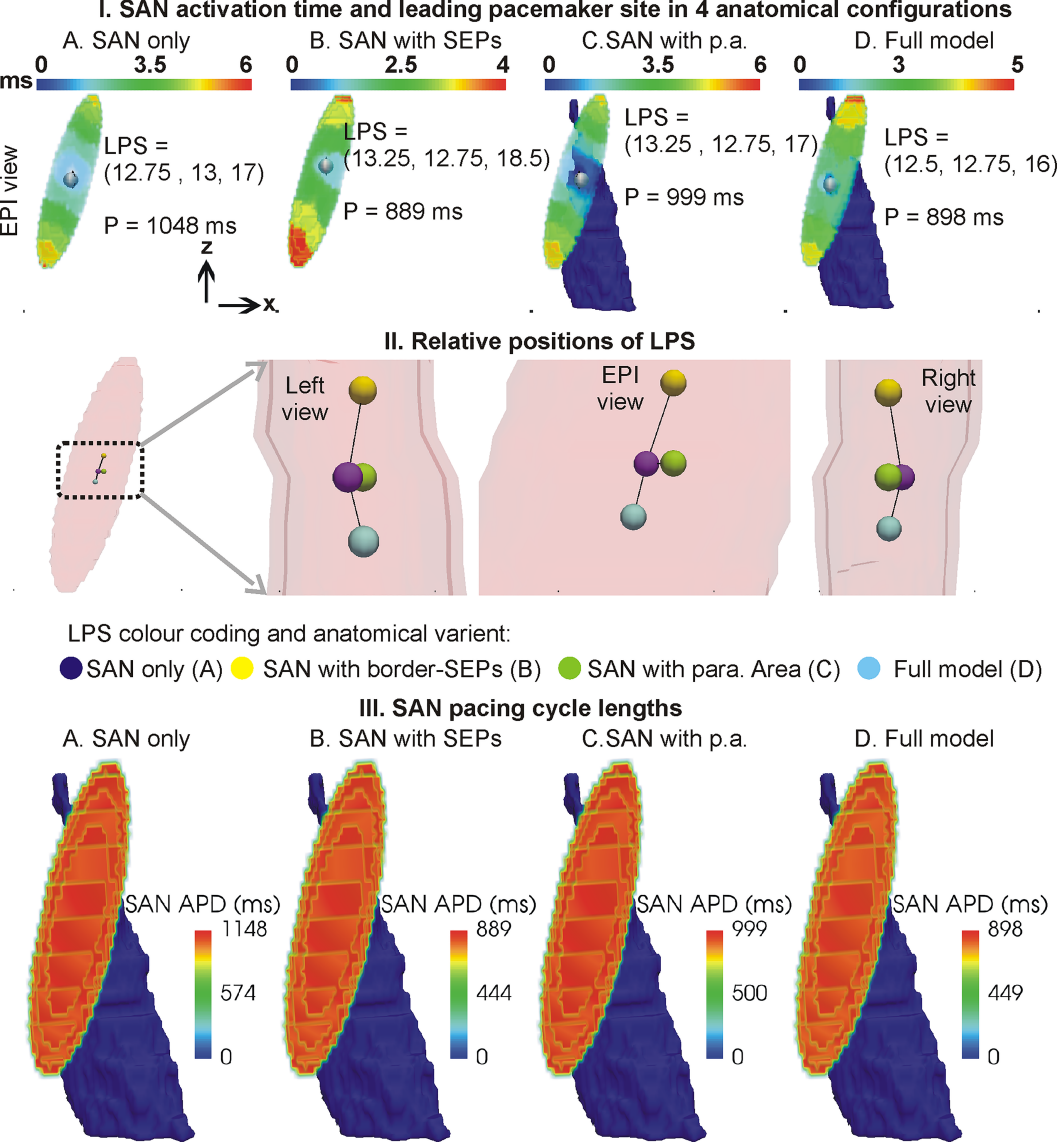
SAN activation times and leading pacemaker sites under basal conditions. I, A-D: Colour coded SAN activation times are shown in four cases. Leading pacemaker site’s (white sphere within the SAN) coordinates and period of atrial pacing, P, for each case are shown. Paranodal area (blue in panels C and D) when present is shown. Atrial tissue (Fig 1) and atrial activation are omitted from the panels for clarity. II. Locations of LPS in anatomical variants. The location of minimum diffusion with the SAN is coincident with the SAN only LPS. III. Cycle lengths of individual SAN cells.

The model micro-structure incorporates the cell-cell coupling and is implemented as diffusive electrotonic cell-cell coupling. Within the electrically homogeneous atrial part of the model, the diffusion was adjusted as in previous studies to 0.35 mm^2^/ms which gave a conduction velocity of 0.6 m/s in the human atrium [28, 30]. The paranodal area was also considered to be electrically homogeneous with a constant diffusion of 0.035 mm^2^/ms. A lower diffusion in the paranodal area as compared to the surrounding atrial part permitted the paranodal area to act as a pacemaker in the absence of SAN pacemaking or external pacing. In contrast to the atrial and paranodal tissues, the SAN is both micro-structurally and electrically heterogeneous. Multiple simulations were performed to dissect the effects of SAN electrical heterogeneity from that of the anatomy. To permit physiological SAN pacemaking, two factors were found critical. Firstly, a diffusion gradient was found essential to permit initiation of electrical wave propagation close to the centroid of the 3D SAN (Fig 1D) [4, 6]. The diffusion gradient may be justified since experimental measurements show gap junction protein distributions within the mammalian SAN where connexin 43 (a gap junction channel protein responsible for conduction velocity) is absent in the centre of the SAN but is present in the periphery [10, 31]. Mathematical modelling studies spanning several decades [4, 6, 32] have incorporated diffusion gradients in the SAN based on experimental findings of SAN conduction heterogeneity, and we adopted a similar approach. To establish the diffusion gradient in our model, smaller ellipsoidal surfaces with the same centroid, ellipticity, as well as long and short axes as that of the SAN were constructed. The smallest surface corresponded to the common centroid, whereas the largest surface was identical to the SAN’s surface. Diffusion values were assigned to the SAN grid points based on the size of the individual ellipsoids. The values ranged from 0.00035 mm^2^/ms for the smallest surface, to 0.35 mm^2^/ms (i.e. atrial diffusion value) for the largest surface. The centroid of the SAN with coordinates (12.75 mm, 12.75 mm, 17 mm) was assigned minimal diffusion. Secondly, electrical heterogeneity (Fig 1E) was implemented within the SAN by simulating a uniformly random distribution of pacemaker cycle lengths devoid gradients with a mean of 850 ms. The electrical heterogeneity ensured that consecutive excitations were always initiated at the centroid of the SAN (see Fig 2).

**Fig 3:**
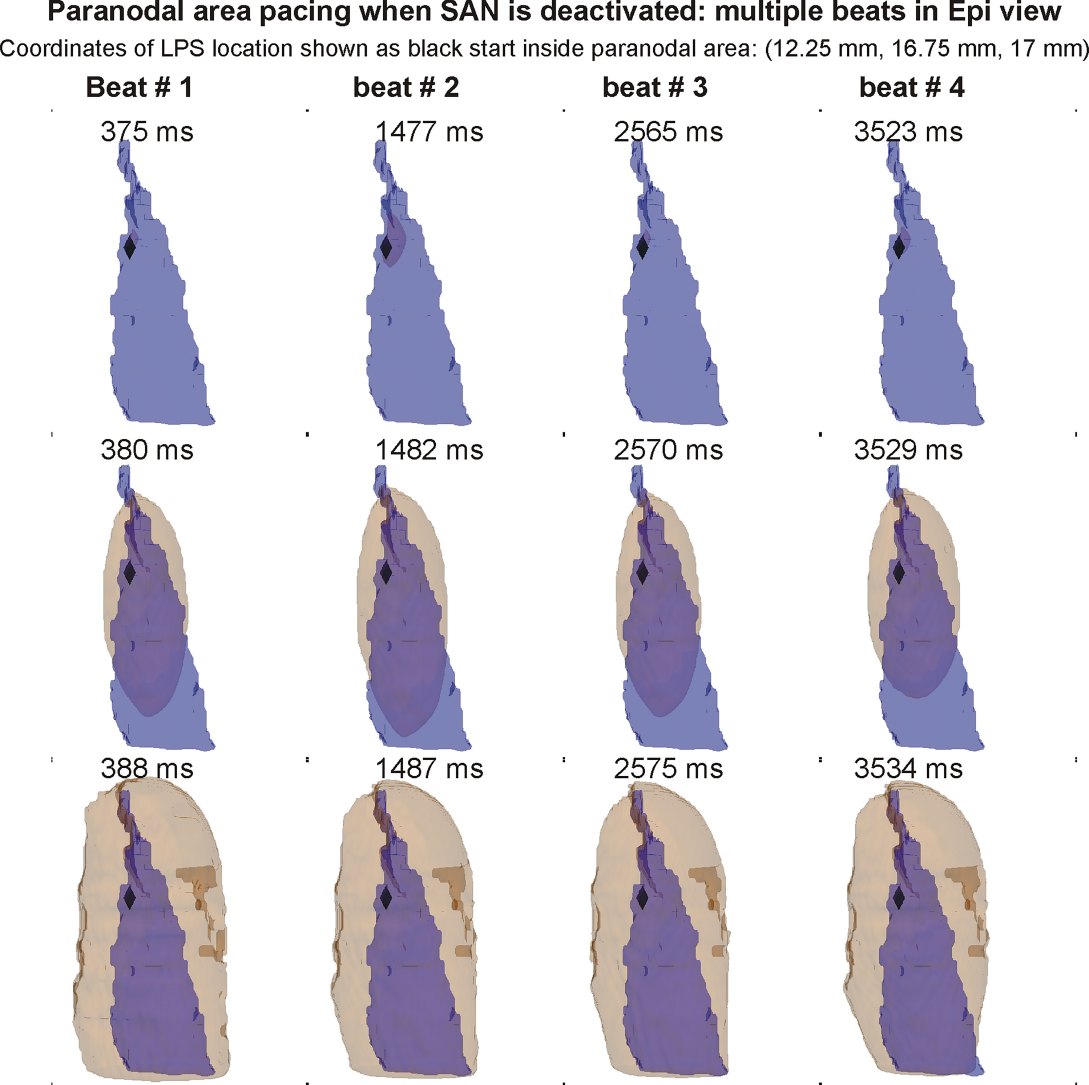
Atrial pacing by the paranodal area. The SAN was replaced by atrial tissue in this simulation. The paranodal area (blue) with activation wave fronts (beige iso-surfaces) are shown, but the surrounding atrial tissue is omitted from the panels for clarity. Top row shows epicardial view, whereas bottom row shows endocardial view. The leading pacemaker site (black star) is within the paranodal area as shown in the snapshots. The period of atrial pacing was found to be 1564 ms.

Unlike the SAN, the paranodal area (as shown in Fig 3) had homogeneous electrical and isotropic diffusion properties and it is a column of tissue that vertically spans the 3D model.

#### 2.1.4 Action of pharmacological agents

The actions of isoprenaline and acetylcholine were qualitatively adapted from experimental data [17, 20]. In those studies [17, 20], both isoprenaline and acetylcholine reduced atrial action potential duration. Therefore, a short action potential of 190 ms was implemented to simulate the actions of isoprenaline or acetylcholine in the atrial cell type. Under the action of isoprenaline the mean pacing cycle of the 3D SAN was arbitrarily reduced to 600 ms, whereas under the action of acetylcholine it was increased to 1200 ms. In this manner, the qualitative action of the two biochemicals was captured in the model. The parameter values simulating the electrophysiology are given in the **S1 Table,** and the action potentials are shown in **S1 Fig.**

### 2.2 Simulation methods

Modifications to the basal anatomy were combined with specific electrical initial conditions to perform a number of simulations. All simulations were executed to produce 5 s of electrical activity in the 3D model according to the mono-domain reaction-diffusion partial differential equation:

$$\partial V/\partial t=\nabla D(x,y,z)\nabla V+I_{ion}$$ Eq 1

Where V is the membrane potential of cell at location *(x*, *y*, *z)*, *D* is the spatially dependent diffusion, and *I*_*ion*_ is the reaction current produced by cell at that location. At the boundaries and at the interface between active and inactive tissue, no flux boundary conditions were implemented, i.e. *D*(*x*,*y*,*z*)∇*V* = 0.

In case of the SAN and paranodal area pacemaking simulations, the model’s electrophysiology was initialised to resting state for the atrial cell types, or minimum potential in case of the pacemaker (SAN and paranodal area) cell types. The system was then permitted to evolve. In simulations demonstrating the role of paranodal pacemaking, the SAN as well as the border-SEPs were replaced with atrial tissue and 5 s of electrical activity simulated. In the case of re-entry simulations, the electrophysiological initial conditions were produced using the phase distribution method as described in detail previously [33, 34]. The phase distribution method permits the initiation of scroll waves at a chosen location. Accurate estimation of the 3D filament locus was achieved using the phase singularity method was used [35]. The 3D phase singularity detection algorithm is illustrated in **S3 Fig.**

### 2.3 Micro re-entry simulation

To identify the micro-structural alterations that are necessary to simulate persistent micro re-entry, the 3D model was altered to incorporate the experimentally observed action of isoprenaline, which was a shorter SAN pacing rate. Re-entry was induced within the SAN using the phase distribution method as described above. The action potential alterations, however, alone were insufficient to permit the inducing of persistent micro re-entry in the 3D model. It is known that non-conducting fibrosis regions can alter conduction patterns and assist in preserving re-entrant waves [36].

**Fig 4:**
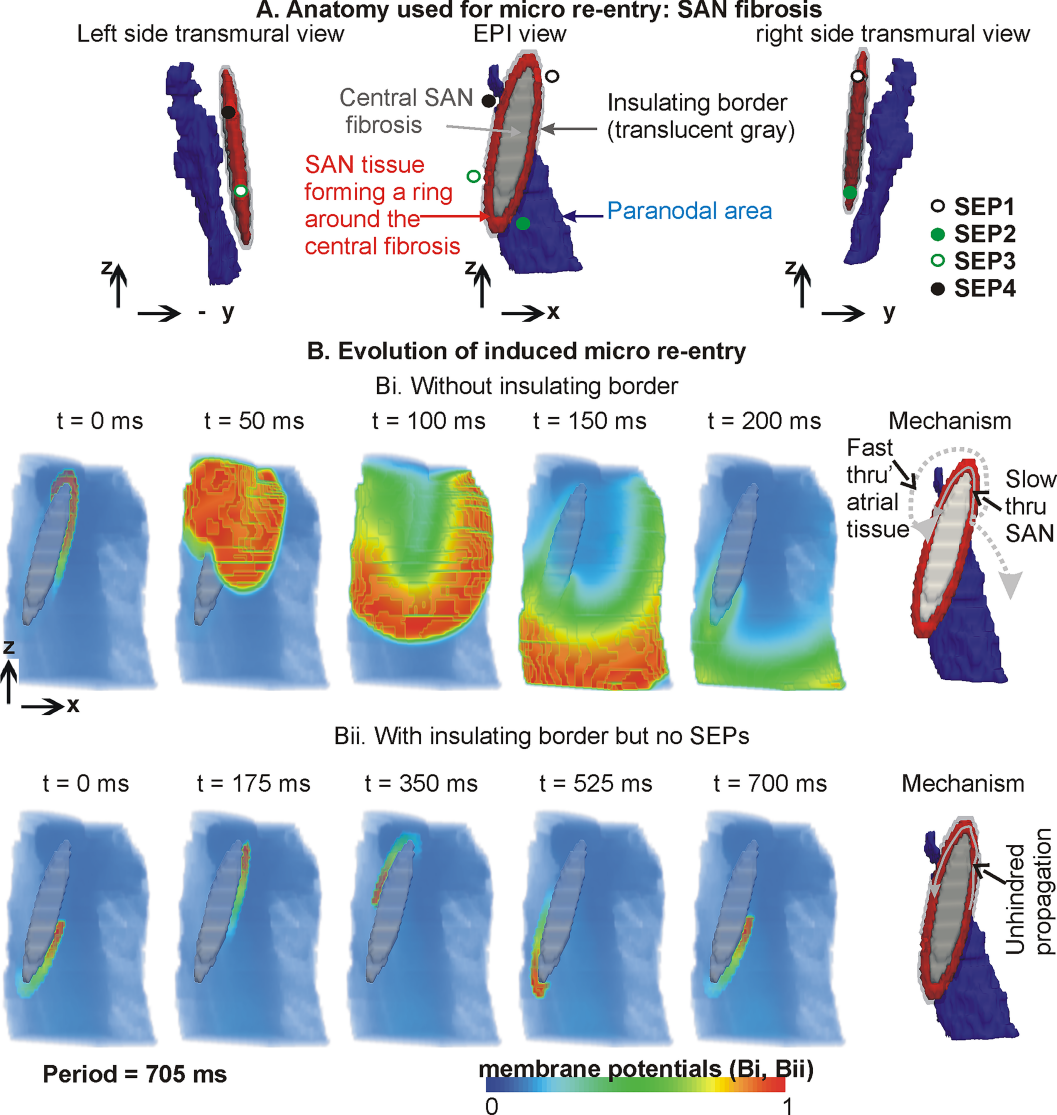
SAN micro re-entry without SEPs. A: Epicardial as well as transmural views of SAN (red), paranodal area (blue), and non-conducting intra-SAN fibrosis (gray). The morphology of the fibrosis is further illustrated by the transmural left and right views. Locations of the omitted SEPs are shown by circular symbols to provide further anatomical landmarks. B: Evolution of micro re-entry. Top row (Bi) shows data from simulation without insulating border. Bottom row (Bii) shows data from simulation with insulating border without SEPs. Bi data show dissipation of re-entry due to absence of insulating border. Sixth panel illustrates the mechanism. Bii shows data with the insulating border configuration where the re-entry persisted unhindered. Sixth panel illustrates the mechanism.

**Fig 5:**
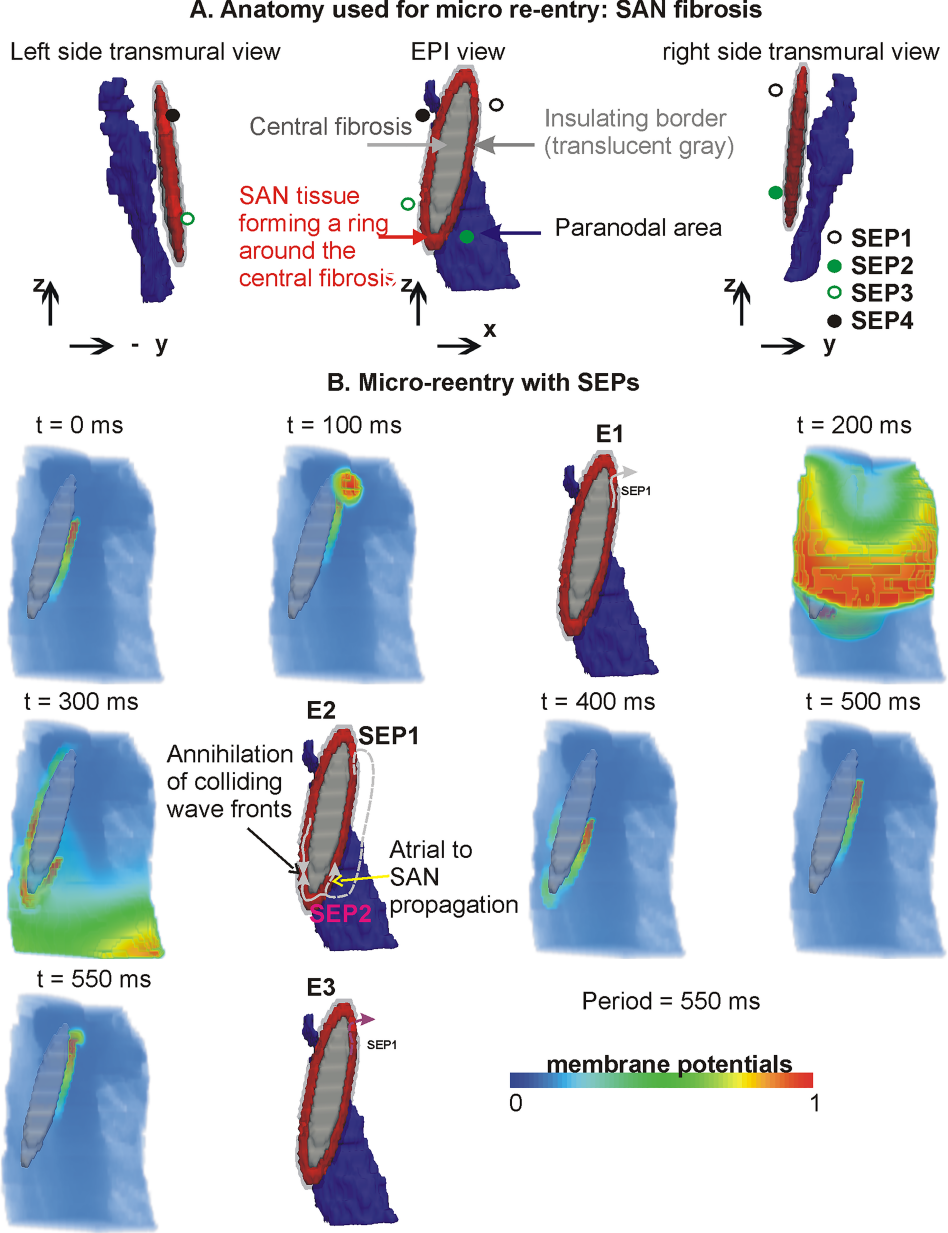
Evolution of micro re-entry in the full model with SEPs. A: Anatomy (similar to Fig 4A) of the SAN, SAN fibrosis, and locations of functional SEPs. B: Consecutive time frames of the micro re-entry in terms of voltage distribution interspersed with panels illustrating the important events that occur during one period of the re-entry. E1 illustrates the SAN propagation initiating excitation in the atrium. E2 illustrates the directions of propagation from the atrium into the SAN, as well as the annihilation and continuing of the SAN re-entry. E3 marks the completion of one period of the re-entry.

Arrhythmogenic cardiac fibrosis is now accepted to be diffuse, patchy, or compact [19]. In terms of computational modelling diffuse fibrosis may be thought as individual cell locations becoming inactive (spatial size much less than 1 mm), while patchy fibrosis would be inactive patches covering regions or strands of around 1–5 mm, and compact being a significantly larger region. The experimental literature suggests that the SAN has interstitial fibrosis strands with millimetre sized dimensions [18, 37]. Modelling results by ten Tusscher and Panfilov [38] suggest that persistent re-entry is more likely under patchy fibrosis rather than diffuse fibrosis conditions. Further, the same group have shown that the propensity of re-entry is significantly higher when the size of the spatial heterogeneity is large (several mm) [39]. Studies from the Vigmond group demonstrate that the spatial size of the heterogeneity is related to the global size of the model [40, 41]. The studies from the both groups show that the spatial extent of the fibrosis is of millimetre scale. Whereas there is a growing literature that has quantifies properties of fibrosis in spatially extended systems of excitable cardiac cells, there is a lack of similar studies that address the same questions in systems of coupled pacemaker cells such as the SAN. Importantly, the studies simulating fibrosis as mentioned above distribute the fibrosis patches either randomly or using imaging data. While imaging data for the SAN fibrosis was unavailable to our study, using random distributions in a 3D model posed challenges in terms of computational costs. Firstly, multiple simulations at a given level of randomly distributed fibrosis would be required. This would be combined with estimating the size-proportion of fibrosis relationships in the SAN which is unknown. Importantly, as the size of the SAN-atrial junction representing SEPs is also uncertain, assessment of the interplay between fibrosis size-SEPs size would add to the computational cost. Finally and most significantly, our goal was to demonstrate whether one instance of fibrosis that permitted micro-reentry. Exploration of SAN fibrosis is out of scope of this study as well as the subject of future studies. Therefore, a simple form of fibrosis was hypothesized to reproduce micro-reentry as illustrated in Fig 4A. The hypothesised fibrosis patch within the SAN was modelled as a short elliptical column to provide a quasi-2D narrow conducting band of SAN tissue as a wave propagation pathway. It permitted electrical wave propagation in the X-Z plane within the available SAN tissue (Fig 4A), but not in the transmural Y-Z plane. Two cases based on the presence and absence of the border were simulated (see Figs 4 and 5 respectively, for model geometry).

### 2.4 Macro re-entry simulation

**Fig 6:**
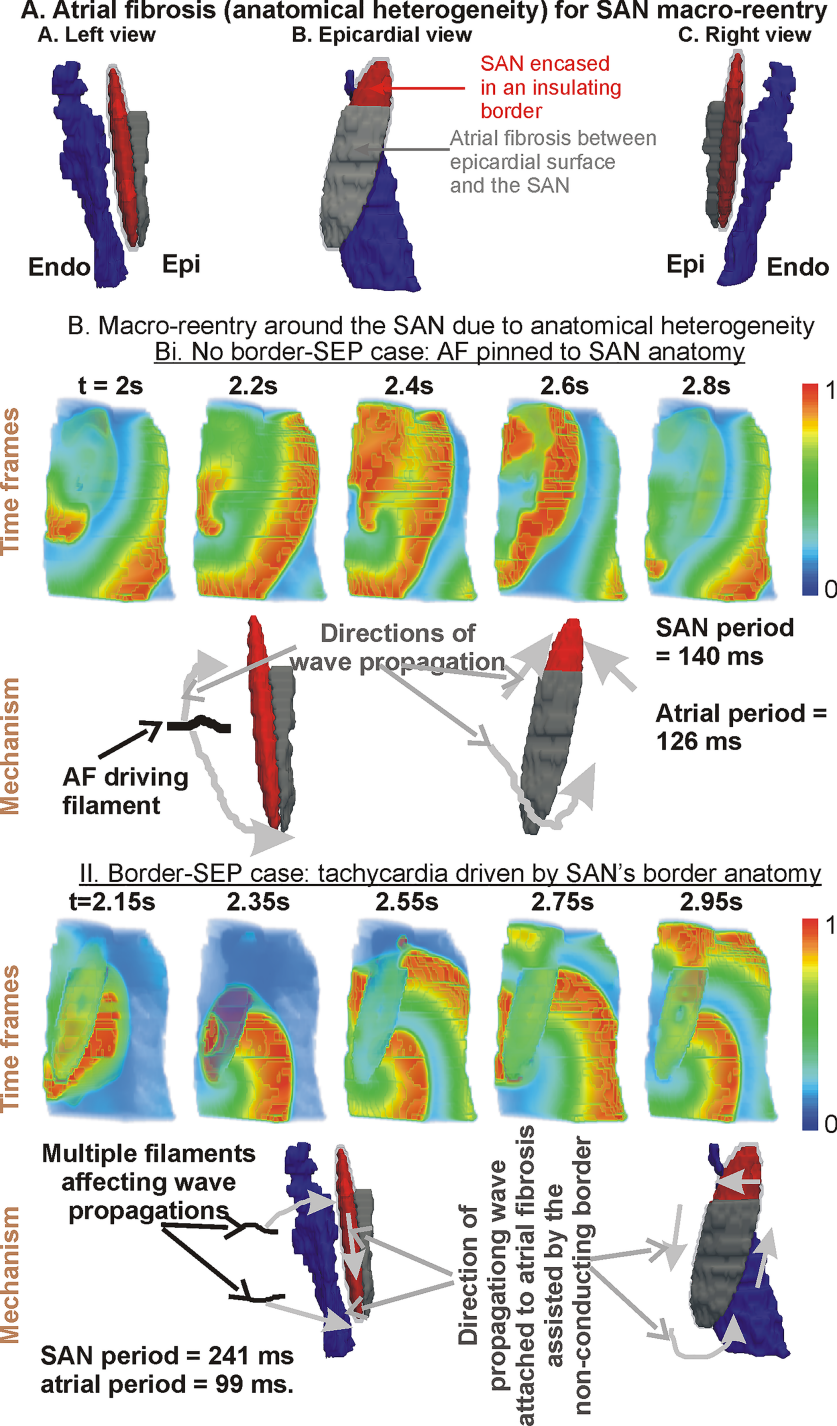
Macro re-entry around the SAN. A: Modified 3D model with atrial fibrosis between the SAN and epicardial surface. B: Simulation of macro re-entry. Bi shows data where insulating border was omitted, where the initiated macro re-entry induced atrial fibrillation. Bii shows data when insulating border-SEPs configuration was included where atrial flutter was observed. In each, Bi and Bi, top row shows time frames from the simulation and bottom row illustrates the mechanism. The periods for SAN and atrial pacing in each simulation is also given.

Another form of re-entry, i.e. circulating waves on the exterior of the SAN, has been observed in experiments [18]. The circulation termed as macro re-entry was observed when the SAN pacemaking was suppressed using acetylcholine. In accordance with the experimental information, the model atrial tissue’s action potential was reduced, which accommodated a re-entry in the atrial part around the SAN. Although SAN action potential was also altered in the experimental set up [18], we first simulated macro re-entry without altering SAN electrophysiology. When macro re-entry was initiated around the SAN, electrical waves propagated around the SAN as well as into the available atrial tissue. This hindered the re-entrant wave’s anchoring to the SAN’s exterior. Therefore, we hypothesised that the myocardial infarction may have induced atrial fibrosis. Transmural atrial fibrosis was found to provide a strong micro-structural substrate for the re-entrant wave anchoring. However, we sought the minimal micro-structural alteration that permitted macro re-entry. It was found that inclusion of fibrosis in the atrial region between the SAN surface and the epicardial surface as illustrated in Fig 6, A may provide the necessary substrate.

### 2.5 Atrial tachycardia initiation

**Fig 7:**
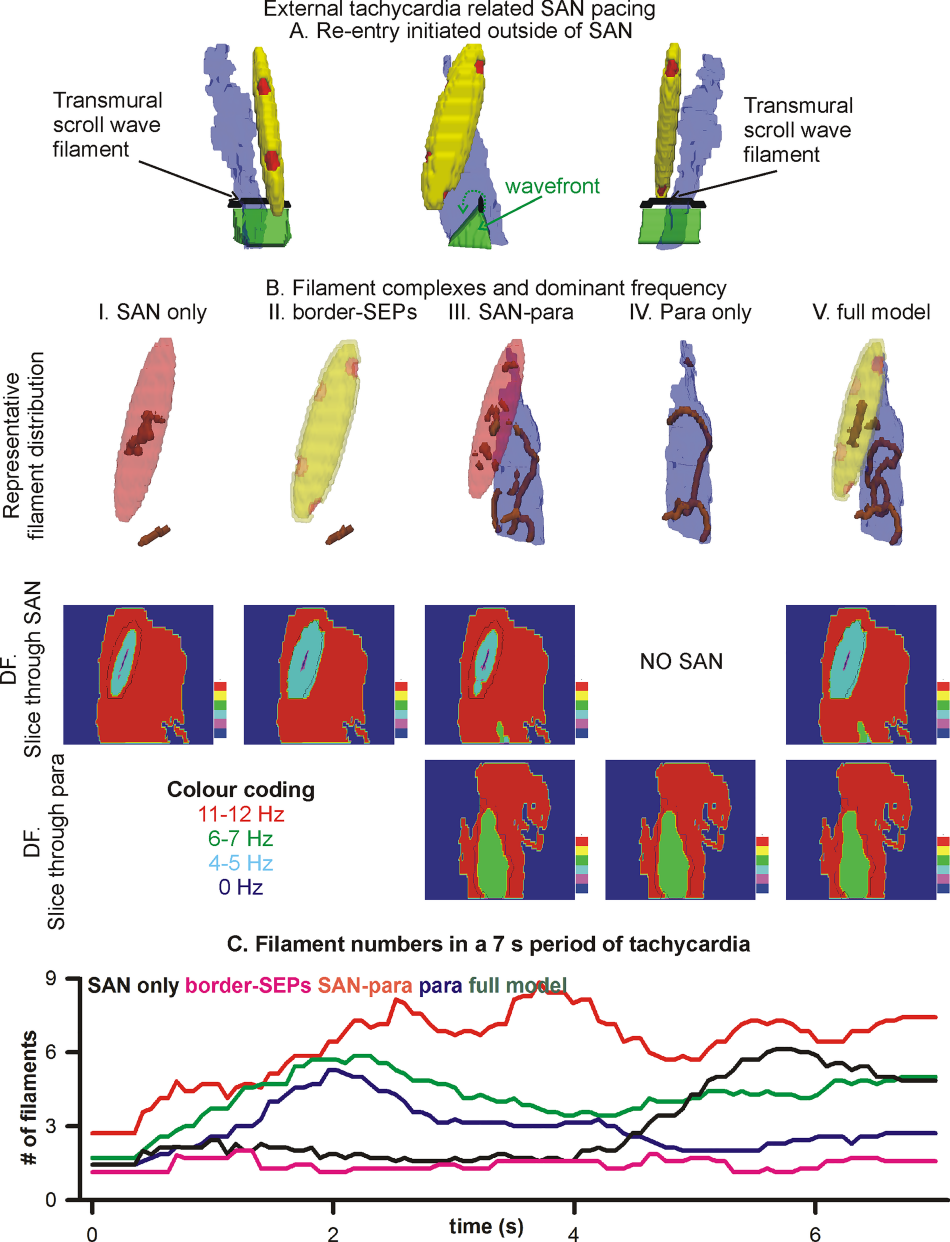
External re-entry. **Effects of atrial tachycardia on SAN excitation.** A: Transmural and epicardial views of the initiated scroll wave (green isosurfaces) and the transmural filament (black line) of the scroll wave. Whereas the full model is shown, 5 anatomical cases were simulated. B: Data for 5 anatomical cases in five columns. The atrial anatomy is omitted for clarity. First row shows representative number and shape of filaments. Second and third rows show dominant frequency (DF) maps of 5 s of data. Slices through the SAN (second row) and through the paranodal area (third row) are shown. C: Time course of filament numbers. The data were smoothed by moving window averaging for clarity.

Atrial tachycardia was induced as a transmural scroll wave using the phase distribution method. The initial transmural scroll wave’s filament was placed at a location where SAN or border tissue was not present while traversing from epicardial to endocardial surface (Fig 7A).

### 2.6 LPS shift simulation

**Fig 8:**
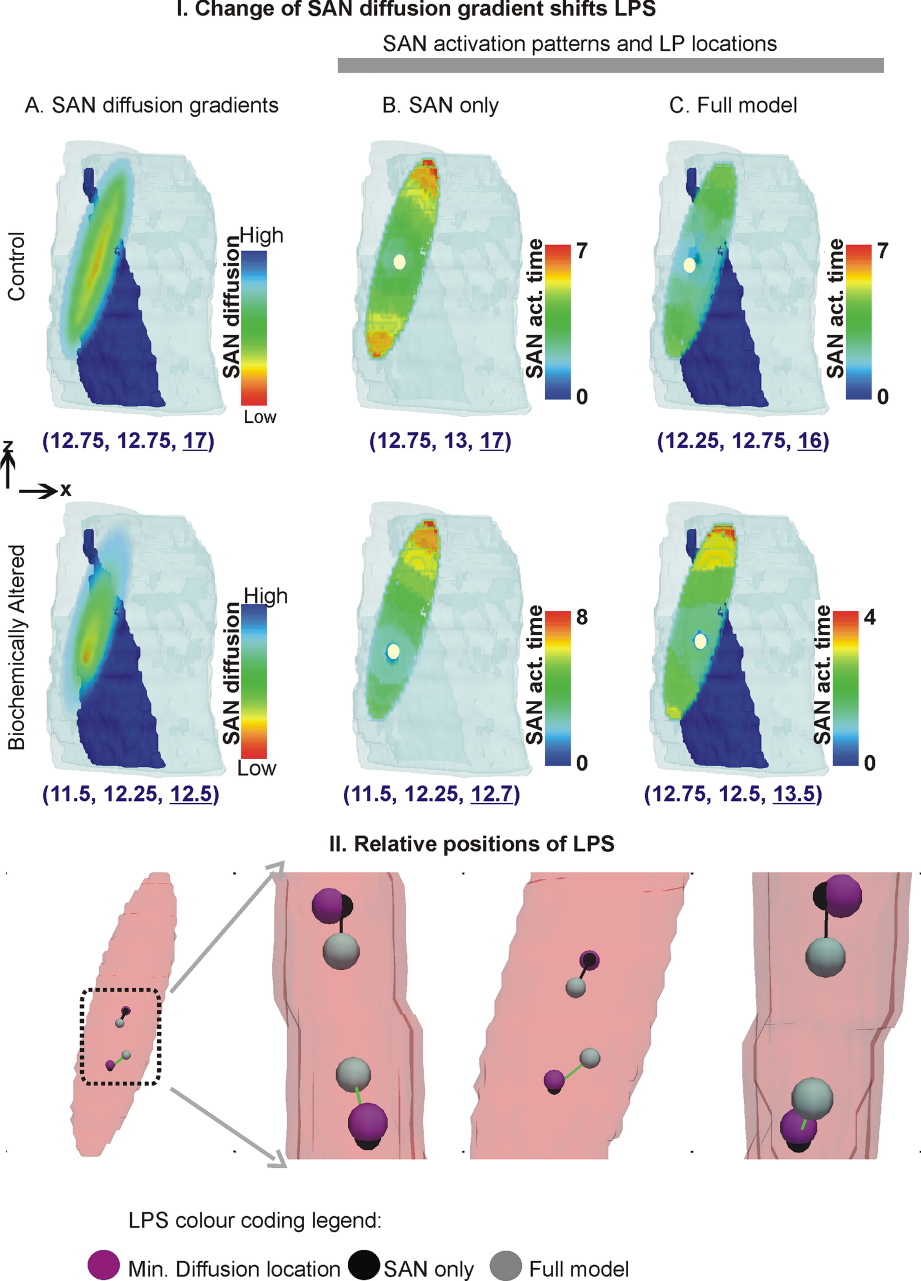
Shift of leading pacemaker site due to altered SAN micro-structure. I: Top row shows data for the control case (Fig 2) while bottom row shows data for the altered SAN micro-structure case. A: model anatomies with colour coded SAN diffusion distribution. The location of the lowest cell-cell coupling is given, which lies within the SAN. B: LPS location in the SAN only case where border was omitted. C: LPS location in the full model case. In columns B and C, the white dots represent location of the leading pacemaker sites while the SAN activation time is colour coded as denoted by the colour bars. The coordinates of the leading pacemaker location are given below the respective panels. II: Vector diagram of the leading pacemaker locations, and locations of minimum diffusion gradients. The minimum diffusion gradients (purple spheres) are joined to the LPS of SAN only (black spheres) and full model (gray spheres). The control LPS are above the biochemically affected LPS.

The location of minimum diffusion (diffusion representing cell-cell coupling) was then shifted to an arbitrarily different location within the SAN, which represented one site of release of the biochemicals from nerve endings. A new diffusion gradient from the new low diffusion location towards the atrium was set up (Fig 8, bottom row, first column).

### 2.7 Numerical methods

The operator splitting method [42] was used to generate numerical solutions of Eq 1 in the 3D model. Operator splitting permitted efficient calculation of the effect of the cell model ordinary differential equations (ODEs) part (**S1 Section**), followed by the PDE part of Eq 1 at each time step. The ODEs were solved using an O(dt^5^) implicit backward difference formula [43]. The PDE part was first discretised using a second order, O(dx^2^), Crank-Nicolson finite difference (FD) formulation. The boundary conditions were incorporated after implementing the phase field relaxation method [44] into the implicit solver to ensure accuracy of the numerical scheme. At each time step, the systems matrix of the PDE was preconditioned and the solution obtained recursively [45]. Whereas both the ODE and PDE solvers exploit the advantages of adaptive time stepping, a user defined maximum time step of dt = 0.1 ms was specified to limit errors. Test simulations at dt = 0.05 ms gave virtually the same results as obtained with dt = 0.1 ms. The solver utilizes MPI based geometric box partitioning to parallelize simulation runs. A simulation run of the model generated 5 s of electrical activity using 48 CPUs for 6 hours. Data in the form of spatial distribution of voltage was recorded at each 1 ms interval to permit post-processing, visualization, and data analysis. Similar to the numerical calculations, data I/O in our code is also parallel thus improving run time efficacy. The solvers and algorithm implementations that have been developed for this study in our laboratory are part of the toolbox providing a new computational dimension to complement our experimental research into the cardiac conduction system.

## Results

### 3.1 Baseline model SAN behaviour with and without SEPs

Activation patterns in the 3D model’s variants within the SAN are illustrated in Fig 2. The activation time of the SAN was found to be between 4 to 6 ms in the four cases (Fig 2I). Leading pacemaker sites (LPS) in all four anatomical variants were located in the vicinity of the SAN’s centroid (12.75 mm, 12.75 mm, 17 mm), as identified by the coordinates of the location within the SAN where electrical propagation was first initiated during any particular heartbeat (Fig 2I). The LPS was seen to be marginally affected by the border-SEPs and the paranodal area, as well as the electrical heterogeneity in the SAN. The atrial pacing rate was mainly affected by the insulating border. Relative locations of LPS under the four anatomical configurations considered are illustrated in Fig 2II. In the absence of the border, the atrial tissue was paced at a period of 1048 ms and 999 ms (Fig 2IA and 2IC respectively). However, in the presence of the border, the atrial tissue experienced a pacing period of 889 ms and 898 ms (Fig 2I, 2BI and 2D respectively). The synchronous firing of all SAN cells was confirmed as shown in Fig 2III. The APD of individual cells throughout the SAN was measured. It was found that all cells fired synchronously at the pacing rates shown in Fig 2III.

### 3.2 Paranodal area pacemaking when SAN is inactive

The activation sequence with the functional paranodal area is shown in Fig 3. The LPS was found to be located within the paranodal area (Fig 3). The paranodal area’s LPS was found to be stable over the duration of the simulation, as shown by its location during several beats. The initiated wave's speed was slower in the paranodal area as compared to the surrounding atrial tissue. This is because its diffusion is 10 fold less than the atrial tissue. In contrast to the short SAN activation times (4–6 ms) as observed in Fig 2, paranodal area activated slowly (15 ms activation time). As individual paranodal area cells have a long cycle length of 1400 ms, the paranodal tissue paced the 3D model at a cycle length of 1565 ms.

### 3.3 Micro re-entry based on SAN fibrosis, no SEPs within insulating border

The anatomical configuration with SAN fibrosis used to simulate micro re-entry is shown in Fig 4A. Unidirectional propagations were initiated in the band of SAN tissue between the SAN fibrosis and atrial tissue. In the case when an insulating border was absent (Fig 4Bi), the induced unidirectional propagation propagated along the fibrosis patch and also into the atrium. Due to the propagation velocity being higher in the atrial part as compared to the SAN, the SAN re-entry dissipated by propagating into the atrial tissue. The mechanism of micro re-entry dissipation is illustrated in Fig 4Bi (last panel). In the case when an insulating border was incorporated, SEPs were excluded to highlight wave dynamics between the fibrosis patch and the insulating border (Fig 4Bii). The presence of an insulating border prevented atrial excitation, thereby permitting the induced unidirectional excitation to propagate unhindered along the available narrow band of SAN tissue. Due to a balance between the isoprenaline induced short propagation wavelength, and the sufficient diffusion in the narrow conducting band of SAN, the micro re-entry persisted almost periodically throughout the 5 s of simulated activity (period = 705 ms). The observed APD during the micro re-entry was 635 ms, and the conduction velocity in the narrow layer of viable SAN tissue was 0.043 m/s, as compared to 0.4 m/s in the atrial part. This gave a wavelength of approximately 27 mm for the circulating wave in the SAN’s viable tissue.

### 3.4 Micro re-entry based on SAN fibrosis, full model with SEPs

The function of SEPs in micro re-entry was then assessed. The paranodal area played a minimal role in the re-entry dynamics. Therefore simulations were performed in the full model (Fig 5). Depending on where the unidirectional propagation was induced, the SEP toward which the excitation was propagating permitted excitation of atrial tissue. Fig 5 shows an instance where the unidirectional propagation moved towards SEP1, and caused excitation of atrial tissue at SEP1 (Fig 5E1). This gave rise to propagations in two distinct directions: within the SAN along the narrow SAN band, as well as in the atrial part where the propagation moved significantly faster. The SAN propagation continued to circulate within the SAN. Whenever it was in the vicinity of a SEP, depending on the repolarisation status of adjacent atrial tissue, it initiated atrial propagation. On the other hand, the atrial propagation moved rapidly through the homogeneous atrial part. When an atrial propagation was in the vicinity of a SEP, it initiated propagation into the SAN depending on the SAN tissue’s repolarisation status. Such an event is illustrated in the t = 300 ms and E2 panels. As shown in Fig 5E2, the atrial propagation that entered the SAN propagated in each direction possible. In the case when propagations collided, they extinguished each other. The unhindered propagations continued in a re-entrant circuit around the fibrosis patch within the SAN. The present model may be inadequate to simulate re-entry using diffuse fibrosis. Therefore, a large central “lump” of fibrosis within the SAN was implemented to render the re-entry persistent (period = 550 ms). In the simulation result shown in Fig 5, the re-entry initially propagates in the SAN tissue between SEP2 and SEP1 counter clockwise. As it transverses SEP1, atrial tissue is stimulated to produce an atrial propagation simultaneous to re-entry propagation within the SAN continuing towards SEP4. When the SAN propagations wave front reached SEP4 or SEP3, the atrial tissue was either already depolarised or refractory. As a result, further excitation of atrial tissue by the propagating SAN excitation did not occur. However, the atrial excitation reached the atrial side of SEP2 prior to the SAN re-entry reaching it from within the SAN. This caused the atrial wave to enter the SAN through SEP2 which propagated in both clockwise and counter clockwise directions. The clockwise wave collided with the prior counter clockwise re-entry. The newly induced counter clockwise propagation continued to propagate along the narrow SAN path between the fibrosis and insulating border region.

### 3.5 Macro re-entry anchors around atrial fibrosis

In the first simulation (Fig 6Bi), re-entry was induced in an anatomy omitting the border. In addition, The SAN-atrial electrical heterogeneity was seen to give simultaneous slow-fast propagations in the vicinity of the SAN. As the time frames of Fig 6Bi show, the combination was sufficient to sustain re-entry that was apparently around the SAN. In this case, the SAN was activated at the same period as the re-entry’s period. The circulation was sustained by the rotor filament (Fig 6Bi, mechanism) being almost stationary in the atrial-paranodal area part from the SAN to the endocardial surface. The arms of the re-entry generated propagations around. The period of the SAN excitations was approximately 150 ms, which was similar to that of atrial excitations was 126 ms. Since the period of the macro re-entry was much less than that of the SAN’s intrinsic pacing period (~ 1 s), the SAN was overdrive suppressed by the circulating waves. In the next simulation, the macro re-entry was induced in an anatomy that included the border-SEPs configuration (Fig 6Bii). Due to the presence of the insulating border, the non-conducting region consisting of the atrial fibrosis and insulated SAN was significantly larger which facilitated circulation of the excitation waves around the SAN. When the waves in the atrial tissue were in the vicinity of SEPs, propagations entered the SAN at those SEPs depending on their repolarisation status. Thus, as the macro re-entry progressed, propagating waves were also initiated inside the SAN through some of the SEPs (e.g. Fig 6Bii, time frames panel for t = 2.35 s). The filament dynamics had a complex pattern (Fig 6Bii, Mechanism). The single initiated filament broke into two or more filaments. Each of the filaments gave rise to propagations in the atrial tissue that contributed to the macro re-entry. The period of the SAN excitations was approximately 241 ms, which was significantly slower than that of the re-entry related rapid atrial excitation period of 100 ms.

### 3.6 Shielding of SAN from external re-entry by SEPs

The evolution of re-entry initiated fully in the atrial region was simulated (Fig 7). The rapid atrial tachycardia overdrive suppressed the SAN’s and paranodal area’s inherent electrical activities in all respective cases. When the border-SEP and paranodal area (Fig 7, SAN only) were omitted, the arm of the tachycardia periodically initiated excitations in the SAN. Due to the electrical SAN-atrial heterogeneity, these excitations were erratic. The wave propagations within the SAN also broke up producing daughter rotors that contributed to the SAN’s erratic pacing. The dominant frequency map shows that the atrial tachycardia caused SAN periphery to be paced at a much higher rate than the centre of the SAN. The filament of the mother rotor meandered due to the erratic activations. Eventually, there were a significant number of small wavelets showing atrial fibrillation.

In contrast, when the border-SEPs were present (Fig 7, SAN with border-SEPs panels), the rapid atrial tachycardia did not pace the SAN at such a high rate due to the insulating effect of the border. The SEPs permitted periodic excitations from the atrial tissue into the SAN tissue. The dominant frequency map shows that the SAN was paced at a much lower rate as compared to the case when the border was omitted. The filament was relatively stable, but was also seen to meander to a certain extent. The number of filaments remained low (Fig 7C). In the case when the paranodal area was included (Fig 7, third and fourth column of panels), the paranodal area caused a large prolongation of the mother rotors filament. The filament extended and often broke down, and multiple daughter filaments were generated (Fig 7B, third column). The complex paranodal area-SAN anatomical region where the paranodal area prolonged the filaments, and the SAN’s diffusion anisotropy caused breakup led to rapid genesis of atrial fibrillation, as shown by the filament numbers (Fig 7C). In the full model (Fig 7B, column V), the border-SEPs insulated the SAN from the atria tachycardia but the paranodal area promoted filament prolongation and often break up of filament. This is reflected in the modest number of filaments seen in the full model (Fig 7C).

### 3.7 LPS is shifted by biochemically induced micro-structural alterations

Fig 8 illustrates that the micro-structure may be a significant factor in LPS shift. In the basal model anatomy, the LPS was found to be at the SAN’s centroid barring any small random functional fluctuations (Fig 8, top row). The LPS in the SAN without a border as well as with a border was found to be in close proximity of the hypothetical nerve ending location. In the case when the border-SEP were present (Fig 8, bottom row, column C), the locations of the SEPs appear to affect the LPS location as a secondary effect to the altered micro-structure. As the SAN is small (approximately 2 mm long), the shift could be simulated only in a limited region. The relative locations of the shifts caused by various anatomies are shown in Fig 8II.

### 3.8 Observation of fibrosis in human SAN

**Fig 9:**
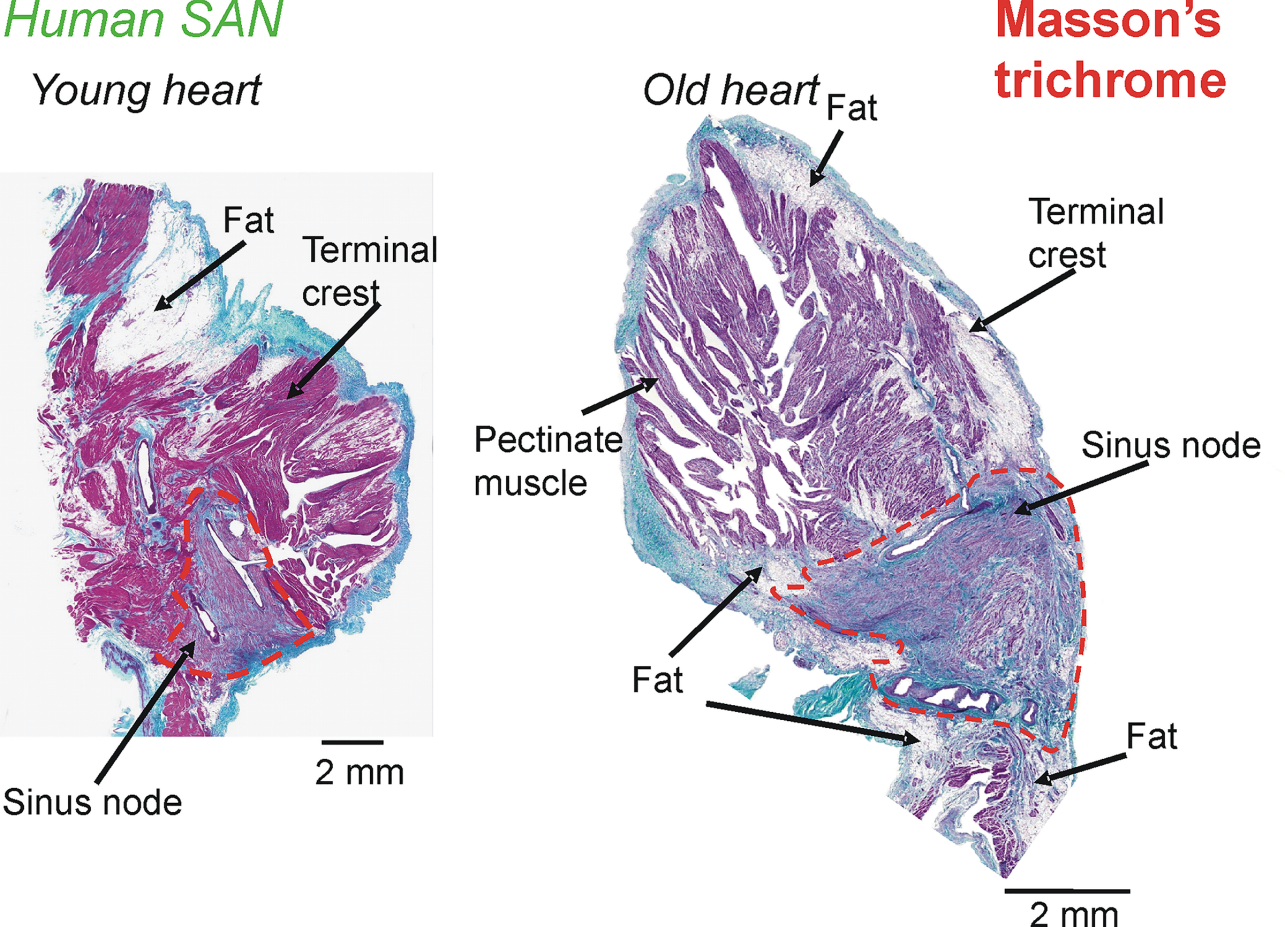
Fibrosis within the old human SAN. The dashed red lines delineate SAN pacemakers. The left panel shows a representative slice from a young heart while the right panel shows a slice from an old heart. The Masson’s trichrome staining shows cell cytoplasm (pink) and connective tissue or fibrosis (blue, blue-green). Whereas the left panel’s SAN region is dominated by pink staining, the right panel has diffuse fibrosis.

Experimental evidence of SAN fibrosis is shown in Fig 9. In comparison to the young heart (Fig 9, left) the old heart has significantly more fibrosis. The fibrosis is distributed throughout the SAN. Although the correlation between the level of fibrosis and the anatomical location within the SAN in the old heart’s SAN cannot be conclusively established, the data strongly suggest that fibrosis may disrupt electrical wave propagation. Under suitable distribution of fibrosis, it may be possible to elicit re-entrant tachycardia in such an old heart.

## Discussion

The three main developments and findings of this study were:

1. establishment of a 3D human electro-anatomical model that incorporates new anatomical features of SEPs and paranodal area;
2. that we tested the hypothesis that micro and macro re-entry are possible due to fibrosis; and
3. to propose a hypothesis anchored in extant experimental data that microstructural alterations are sufficient for an LPS shift, and do not require electrophysiological modifications.

### 4.1 De novo 3D human SAN model

To the best of our knowledge, we have presented here the first SAN electro-anatomical 3D model of the human. It incorporates known and hypothesised anatomical features and is capable of presenting plausible arrhythmia mechanisms which can be tested in the laboratory and clinic. The model was used to simulate multiple electrical events to identify prime factors anchored in anatomy and micro-structure.

### 4.2 Baseline model establishment

Baseline SAN pacemaking simulation using our 3D model shows that the micro-structure consisting of a SAN cell-cell coupling gradient regulates LPS location. The assumed random electrical heterogeneity conferred robustness to where the SAN waves originated, it was not found necessary to include a further SAN electrical gradient. Whereas the optical mapping experiments point towards conduction pathways originating from the SAN where they form SEPs, the gradients hypothesis requires a revision in light of the new data [4, 6, 7].

### 4.3 Simulation of observed micro re-entry

In a previous study, micro re-entry has been simulated within a large block of pacemaker tissue in the absence of SAN fibrosis [46]. However, we can appreciate that realistic SAN (Fig 1) is small which may be incapable of containing a rotating scroll wave with a large wavelength. Factors such as intra-SAN fibrosis were found to be necessary to preserve micro re-entry. Indeed, pharmacological agents induce electrophysiological as well as microstructural (i.e. fibrosis) alterations [18]. A large non-conducting region created a pathway for circular propagation in our model. The pathway consists of SAN cells whose excitation depends on a slow diastolic depolarisation. A weaker diffusion as compared to the atrial part combined with the slow diastolic depolarisation of the SAN cells in the pathways generated a conduction velocity of 0.043 m/s in our model, which is comparable to the experimental estimates of 3 to 12 cm/s by Fedorov et al. [20].

Although the hypothesised fibrosis is one example, other SAN fibrosis configurations that promote micro re-entry are also possible [47]. The simulations of Fig 4 indicate that SAN micro re-entry may persist due to its shielding from atrial hyperpolarisation or other atrial excitations. Of course, more numerous small patches of fibrosis may also be a causal factor of smaller re-entrant circuits, as observed in idealised atrial tissue simulation of fibrosis [48]. In addition, in the presence of SEPs, the re-entry within the SAN is associated with complex SAN-atrial electrical interaction promoting a more rapid tachycardia (period = 550 ms) as compared to a re-entry purely circulating around the SAN. From the simulations for micro re-entry, it is clear that an insulating border is necessary for shielding the SAN rapid excitation from dissipating into the surrounding atrial tissue.

### 4.4 Macro re-entry

Similar to micro re-entry, the pharmacological agents and pacing protocols used to induce macro re-entry in the experiments setups may themselves promote fibrosis. In our simulations, we hypothesise that atrial fibrosis between the SAN and epicardial surface provides a sufficient micro-structural substrate for persistent macro re-entry. During macro re-entry, it can be seen that a potential border-SEPs configuration firstly insulates the SAN, but is also a crucial configuration that can explain complex atrial-SAN-atrial propagations. Further focused clinical and experimental examination is required to observe such rapid tachycardia, and also pin point the nature of the border-SEPs anatomy. The atrial fibrosis, however, was crucial in sustaining the atrial flutter. Although the atria induced SAN excitation was sufficiently rapid to suppress the SAN’s intrinsic pacemaking, it can be appreciated that the mechanism of macro re-entry relies on atrial fibrosis. The SAN activation was significantly more periodic in the presence of a border in contrast to the no border case, which is phenomenologically in agreement with the experimental findings [18]. In addition, the existence of a border-SEPs configuration somewhat shields the SAN from external tachycardia.

### 4.5 Shift of LPS due to altered gap junction coupling

Whereas electrophysiological alterations have been implicated in the LPS shift [4, 49, 50], the presented model permitted a new correlation between intra-SAN leading pacemaker site and SAN cell-cell coupling microstructure. Of course, to simulate the shift of leading pacemaker outside of the SAN will require additional components to the presented model which may include sympathetic and parasympathetic stimulation as well as biophysically detailed electrophysiology. In the LPS shift simulation of Fig 8, we demonstrated an additional factor contributing to the shift of LPS location. The mechanism of LPS shift to outside of the SAN may not involve a simple micro-structural alteration, but may have to be accompanied by electrophysiological alterations as well.

## Study limitations

The data presented in this study must be interpreted within the confines of the model limitations, as well as experimental data limitations.

### 5.1 Realistic estimates from detailed electrophysiology

In this study, a simple Fenton Karma model was used to simulate electrical excitation throughout the model’s tissue types. The use of the three variable phenomenological cell models permitted rapid demonstration of the observed experimental phenomena. However, future studies using ionically detailed cell models for the human SAN and atrium are required. The use of ionically detailed cell models will provide improved implementation of pacing rates, action potential durations, take off potentials, and upstroke velocities, and provide better estimates of refractoriness and wavelengths. The detailed electrophysiology will also permit a better implementation of diffusive inter-cellular coupling, which is usually estimated based on observed conduction velocity. As future data for secondary pacemakers such as paranodal area become available, they will be also be incorporated in future studies. We appreciate that detailed cell models of human SAN [8, 51] and atria [52, 53] will further improve the simulation of basal and altered electrical activity due to acetylcholine and isoprenaline to permit a better reproduction of the experimental phenomena of micro- and macro- re-entry. It will also assist in more accurate estimation of SAN and atrial conduction properties in the presented model. The source-sink relationship between the two tissue types [54] in respect of a potential insulating border-SEPs anatomical configuration should also be explored using the detailed cell models. It is thought that the paranodal area acts as a secondary pacemaker in the human heart [14]. In the presented model, we therefore assigned a much slower cycle length to the paranodal area’s cell type as compared to the SAN, to permit the SAN to overdrive suppress the paranodal area during physiological pacemaking. The electrical properties of the paranodal area may be better assessed by use of validated electrophysiological models in tissue types that surround it, i.e. SAN and atrial tissue. However, the phenomena of interest could be simulated using the simple Fenton-Karma dynamics, and the electrophysiological information content of the presented model will be extended in future work.

### 5.2 Anatomical model limitations

A limitation of our model may be that the SAN activation we simulated is 5–10 ms, in contrast to the much longer experimentalist’s observation of 40–80 ms [18, 20]. An important reason for the difference could be that the conduction velocity was far slower in the experimental preparations. Another reason could be that the electrophysiology simulated in this study cannot capture the ion channel detail present in real right atrial preparations. In either case, the conduction velocity and closer matching of modelled electrophysiology to actual SAN preparations will affect the SAN’s pacemaking rate as well as numerical values of periods of re-entry. It may also affect the complexity of the SAN-atrial propagations that have been observed in our simulations.

The locations of SEPs in our model were based purely on diagrammatic representations from past studies in the literature [16, 20] rather than being directly mapped from experimental images onto the 3D anatomy. The locations and inter-SEP distance will affect the dynamics of the simulated re-entrant phenomena in this study, but the overall results are expected to be qualitatively similar. Whereas a biophysically detailed accurate model is under development, the presented model is phenomenological and aimed to establish correlations between SAN anatomy and electrical function. The 3D model’s spatial extent constrained the simulation of more realistic event simulations. In the future, we will incorporate nearby blood vessel ostia that will act as sources of ectopy as well as tachycardia pinning. A larger atrial tissue region will also permit simulation of realistic scroll wave dynamics based on clinically measured action potential durations.

### 5.3 Anatomical anisotropy

Due to the focus of this study being the electro-anatomy, fibre orientation micro-structure was omitted. However, it is expected that fibrosis will affect fibre orientation. In the future, and especially in spatially larger models, it is relevant to incorporate fibre orientation information based on detailed imaging data or theoretical models [3, 55, 56].

### 5.4 Inter-cellular diffusive coupling in the paranodal area and SAN

The study of the paranodal area is yet nascent [14, 51, 57]. To the best of our knowledge, experimental action potential recordings and pacemaking properties as well as conduction velocity estimates are unavailable. This necessitated implementation of realistic but arbitrary diffusion-electrophysiological properties. It may be noted that our choice of parameters for the paranodal within the 3D model permitted the simulation of several experimentally observed SAN complex phenomena.

The diffusion gradient in the SAN region of our model permits pacing of surrounding atrial tissue. The gradient may be the result of altering gap junction protein expression from centre to periphery [10, 31], or due to other factors such as fibroblast heterogeneity [58] both of which regulate conduction velocity. As the effects of gap junctions are summarised by the diffusion in the model, a gradient may be justified as implemented in previous modelling studies. However, it should be noted that the exact mathematical form of the increase of conduction from centre to periphery is yet to be estimated experimentally. Whereas a distance measure has been used in this study, others have used a spectrum of different equations and formulations [4, 6, 32]. Future simulations are required to assess which formulations of SAN conduction heterogeneity permit reproduction of the exit pathway related phenomena. It is also important to assess the critical threshold at which atrial pacing becomes possible, especially within our model where the exit pathways provide a spatially limited electrical coupling between the SAN and atrial parts.

### 5.5 Further investigation for fibrosis validation

In contrast to the experimental studies that demonstrated initiation as well as persistence of micro-reentry [17, 18], the present study focused on one simple anatomical configuration, in terms of a single central SAN fibrosis patch, that could permit persistence of an artificially induced micro-reentry. The limited implementation of fibrosis in our model SAN may explain the differences between our estimates of micro-reentry attributes to those in the experimental studies. However, the nature of fibrosis and its consequences on electrical conduction behaviour is complex as reflected by multiple ongoing computational-experimental studies.

The extensive electro-anatomical investigations undertaken by several experimental groups indicate that atrial fibrosis, and cardiac fibrosis in general, falls in three categories: diffuse, patchy, or compact [19]. The qualitative data relevant to this study indicates that SAN fibrosis [18, 37] is patchy in hearts where SAN micro-reentry could be observed [18, 20]. This observation correlates with known computational results that a patchy fibrosis promotes genesis and persistence of re-entry [38, 39]. The simulation of re-entry due to diffuse fibrosis may occur with a small probability [48] as well as be relatively short lived, unless very specific fibrosis within the whole 3D anatomy conditions prevail [40, 41]. However, it is also known that diffuse fibrosis assists in stabilising re-entrant arrhythmia [38] by causing overall slowing of the propagating wave. In this study, we opted to establish a simple yet robust anatomical substrate for producing micro-reentry. The interplay between hitherto unknown SAN fibrosis patch size, amount of total fibrosis, size of SEPs, and other factors has not been estimated as part of this study, and will form the subject of future computational, or experimental-computational studies. A further factor that is relevant to more fully understand SAN function is the myocyte-fibroblast interaction [59, 60], especially in our 3D model with SEPs.

## Conclusions

The data from this study indicates that the insulating border-SEPs configuration plays a crucial role in regular physiopathological SAN conduction. Inclusion of the configuration into 3D models may help to explain observations from other studies. Further experimental-computational exploration is required to translate the findings for clinical relevance.

The 3D model can be obtained from the authors. The anatomical data used to simulate Figs 1 and 8 is provided as an electronic supplement zip file/online repository.

## Supporting information

S1 Fig Role of modelling parameters (S1 Table) on SAN pacemaker and atrial action potentials. A: Range of SAN action potentials used to simulate basal pacemaking. B: Range of SAN action potentials used to simulate the fast SAN pacemaking, to simulate the effect of ISO. C: Range of SAN action potentials used to simulate the slow SAN pacemaking, to simulate the effect of Ach.
(PDF)

S2 Fig Geometries of SAN segmented from imaging data (top row) and modified ellipsoidal SAN (bottom row). The columns show views in endo to epi transmural (left column), epicardial (middle column), and the epi to endo transmural (right column) directions.
(PDF)

S3 Fig Illustration of the filament tracing method. A: Representative scroll wave in the 3D model. The “X” shows the arbitrarily chosen action potential recording location in the atrial part of the model. B: The recorded action potential at location “X”. Correlation was computed for several values of delay, τ, between voltages at a fixed time, t, and voltages after a delay at time t+ τ. C: Correlation between voltage at time t and time t+ τ of the recorded action potential. The optimal delay between consecutive frames was identified as 15.2 ms from the correlation. D: A phase plot of the 10 s long action potential was used to identify the parameters to be used in computation of phase. V*(t) = 0.509, V*(t+ τ) = 0.59 were identified. E: The colour coding shows the phase of a representative scroll wave between–π and +π. Solid red shows the SAN to provide an anatomical reference to the reader. The phase singularity is shown as the black transmural filament [35].
(PDF)

S1 Section Cell model equations and parameters. (PDF)

S2 Section Objective method for filament tracking. (PDF)

S1 Table Model parameter values in the cell types of human SAN model. Control values are given in black, and ISO (short SAN AP, short atrial AP) as well as Ach values (long SAN AP, short atrial AP) are given in red.
(PDF)
