# Supplementary material for: Computational assessment of the functional role of sinoatrial node exit pathways in the human heart
Source: PLoS One. 2017 Sep 5;12(9):e0183727. doi: 10.1371/journal.pone.0183727 (PMC5584965; doi:10.1371/journal.pone.0183727)
Supplement: S1 Fig — A: Range of SAN action potentials used to simulate basal pacemaking. B: Range of SAN action potentials used to simulate the fast SAN pacemaking, to simulate the effect of ISO. C: Range of SAN action potentials used to simulate the slow SAN pacemaking, to simulate the effect of Ach. (PDF) [file pone.0183727.s001.pdf]

Supplementary Data

Computational assessment of the functional role of sinoatrial node exit pathways in the human heart

Sanjay R Kharche[1*], Edward Vigmond[2, 3], Igor R Efimov[4], Halina Dobrzynski[1*]

[1] Institute of Cardiovascular Sciences, School of Medical Sciences, University of Manchester, Manchester, M13 9NT, UK

[2] University of Bordeaux, IMB, UMR 5251, F-33400 Talence, France

[3] IHU Liryc, Electrophysiology and Heart Modeling Institute, Fondation Bordeaux Université, F-33600 Pessac- Bordeaux, France

[4] Department of Biomedical Engineering, The George Washington University, Washington, DC, 20052 USA

Supplementary Methods

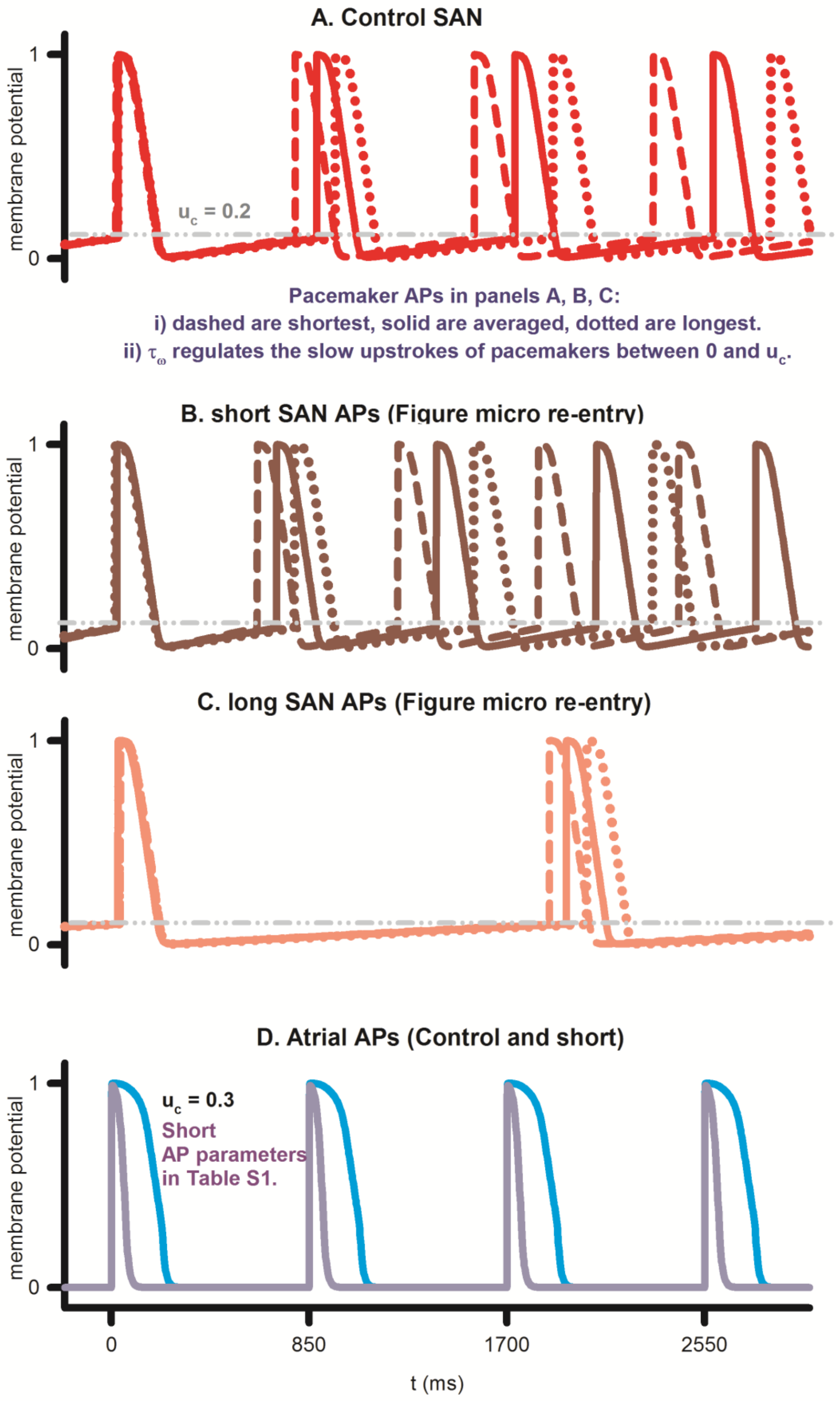

S1 Fig. Role of modelling parameters (Table S1) on SAN pacemaker and atrial action potentials. A: Range of SAN action potentials used to simulate basal pacemaking. B: Range of SAN action potentials used to simulate the fast SAN pacemaking, to simulate the effect of ISO. C: Range of SAN action potentials used to simulate the slow SAN pacemaking, to simulate the effect of Ach.
