# Supplementary material for: Computational assessment of the functional role of sinoatrial node exit pathways in the human heart
Source: PLoS One. 2017 Sep 5;12(9):e0183727. doi: 10.1371/journal.pone.0183727 (PMC5584965; doi:10.1371/journal.pone.0183727)
Supplement: S2 Fig — The columns show views in endo to epi transmural (left column), epicardial (middle column), and the epi to endo transmural (right column) directions. (PDF) [file pone.0183727.s002.pdf]

Supplementary Data

Computational assessment of the functional role of sinoatrial node exit pathways in the human heart

Sanjay R Kharche[1*], Edward Vigmond[2, 3], Igor R Efimov[4], Halina Dobrzynski[1*]

[1] Institute of Cardiovascular Sciences, School of Medical Sciences, University of Manchester, Manchester, M13 9NT, UK

[2] University of Bordeaux, IMB, UMR 5251, F-33400 Talence, France

[3] IHU Liryc, Electrophysiology and Heart Modeling Institute, Fondation Bordeaux Université, F-33600 Pessac- Bordeaux, France

[4] Department of Biomedical Engineering, The George Washington University, Washington, DC, 20052 USA

Supplementary Methods

Comparison between original and modified SAN geometry

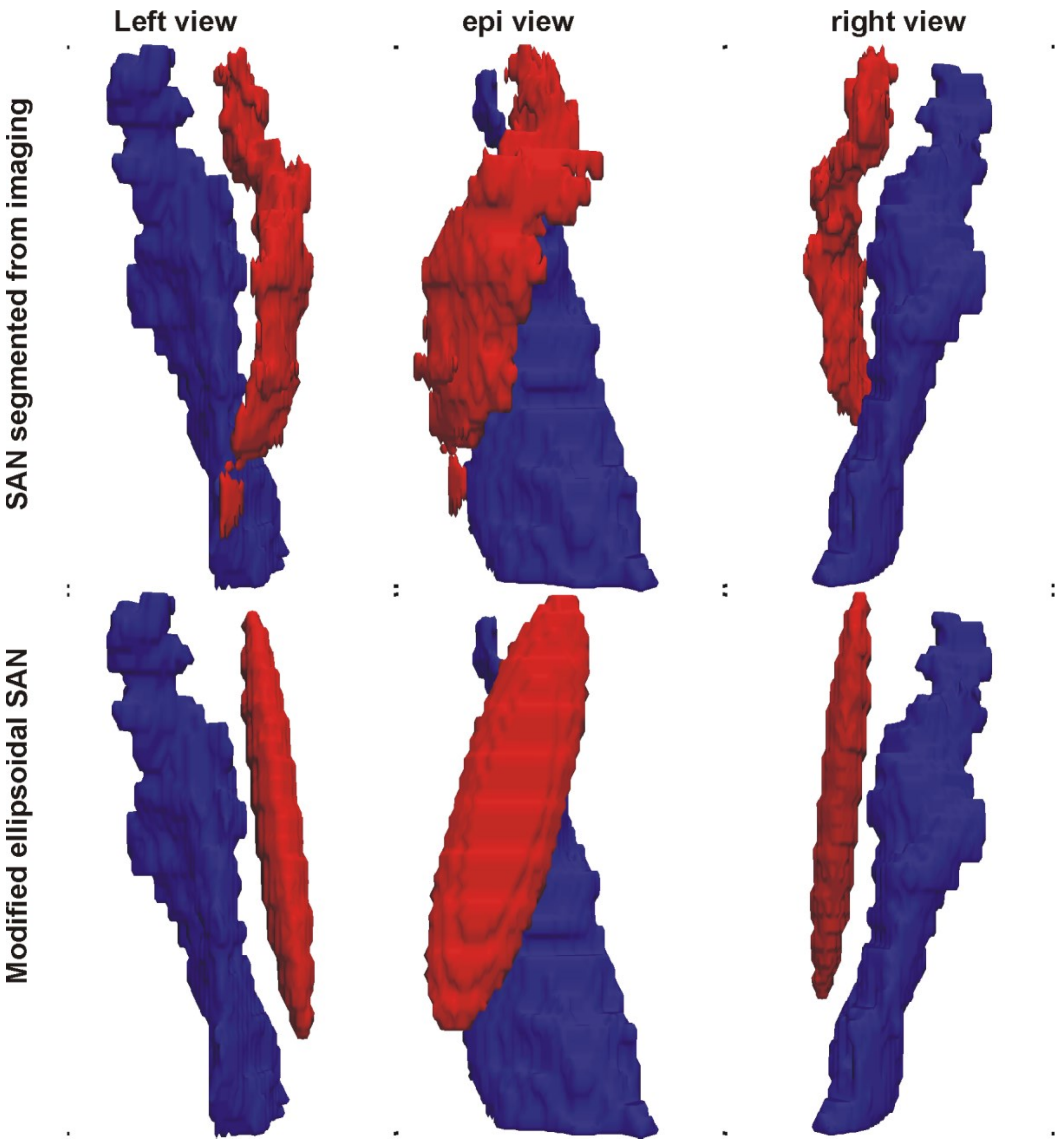

S2 Fig. Geometries of SAN segmented from imaging data (top row) and modified ellipsoidal SAN (bottom row). The columns show views in endo to epi transmural (left column), epicardial (middle column), and the epi to endo transmural (right column) directions.
