# Supplementary material for: Computational assessment of the functional role of sinoatrial node exit pathways in the human heart
Source: PLoS One. 2017 Sep 5;12(9):e0183727. doi: 10.1371/journal.pone.0183727 (PMC5584965; doi:10.1371/journal.pone.0183727)
Supplement: S3 Fig — A: Representative scroll wave in the 3D model. The “X” shows the arbitrarily chosen action potential recording location in the atrial part of the model. B: The recorded action potential at location “X”. Correlation was computed for several values of delay, τ, between voltages at a fixed time, t, and voltages after a delay at time t+ τ. C: Correlation between voltage at time t and time t+ τ of the recorded action potential. The optimal delay between consecutive frames was identified as 15.2 ms from the correlation. D: A phase plot of the 10 s long action potential was used to identify the parameters to be used in computation of phase. V*(t) = 0.509, V*(t+ τ) = 0.59 were identified. E: The colour coding shows the phase of a representative scroll wave between–π and +π. Solid red shows the SAN to provide an anatomical reference to the reader. The phase singularity is shown as the black transmural filament [35]. (PDF) [file pone.0183727.s003.pdf]

Supplementary Data

Computational assessment of the functional role of sinoatrial node exit pathways in the human heart

Sanjay R Kharche[1*], Edward Vigmond[2, 3], Igor R Efimov[4], Halina Dobrzynski[1*]

[1] Institute of Cardiovascular Sciences, School of Medical Sciences, University of Manchester, Manchester, M13 9NT, UK

[2] University of Bordeaux, IMB, UMR 5251, F-33400 Talence, France

[3] IHU Liryc, Electrophysiology and Heart Modeling Institute, Fondation Bordeaux Université, F-33600 Pessac- Bordeaux, France

[4] Department of Biomedical Engineering, The George Washington University, Washington, DC, 20052 USA

Supplementary Methods

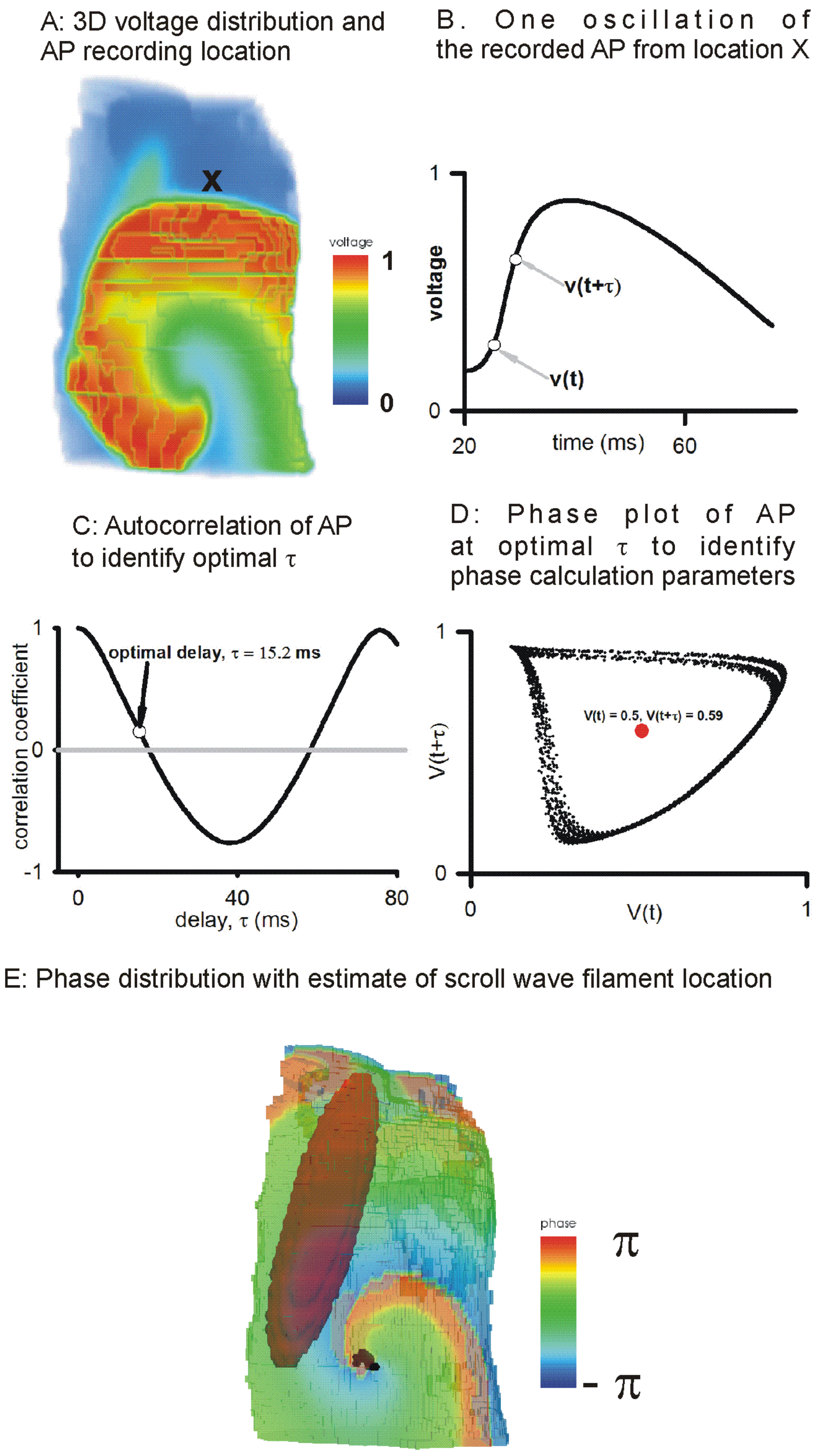

S3 Fig. Illustration of the filament tracing method. A: Representative scroll wave in the 3D model. The "X" shows the arbitrarily chosen action potential recording location in the atrial part of the model. B: The recorded action potential. Correlation was computed for several values of delay, τ, between voltages at a fixed time, t, and voltages after a delay at time t+ τ. C: Correlation between voltage at time t and time t+ τ of the recorded action potential. The optimal delay between consecutive frames was identified as 15.2 ms from the correlation. D: A phase plot of the 10 s long action potential was used to identify the parameters to be used in computation of phase. $V^*(t) = 0.509$, $V^*(t+ \tau) = 0.59$ were identified. E: The colour coding shows the phase of a representative scroll wave between $-\pi$ and $+\pi$. Solid red shows the SAN to provide an anatomical reference to the reader. The phase singularity is shown as the black transmural filament [1].

References

1. Bray MA, Wikswo JP. Use of topological charge to determine filament location and dynamics in a numerical model of scroll wave activity. IEEE Trans Biomed Eng. 2002;49(10):1086-93. Epub 2002/10/11. doi: 10.1109/TBME.2002.803516. PubMed PMID: 12374332.
