# Supplementary material for: Computational assessment of the functional role of sinoatrial node exit pathways in the human heart
Source: PLoS One. 2017 Sep 5;12(9):e0183727. doi: 10.1371/journal.pone.0183727 (PMC5584965; doi:10.1371/journal.pone.0183727)
Supplement: S1 Section — (PDF) [file pone.0183727.s004.pdf]

Supplementary Data

Computational assessment of the functional role of sinoatrial node exit pathways in the human heart

Sanjay R Kharche[1*], Edward Vigmond[2, 3], Igor R Efimov[4], Halina Dobrzynski[1*]

[1] Institute of Cardiovascular Sciences, School of Medical Sciences, University of Manchester, Manchester, M13 9NT, UK

[2] University of Bordeaux, IMB, UMR 5251, F-33400 Talence, France

[3] IHU Liryc, Electrophysiology and Heart Modeling Institute, Fondation Bordeaux Université, F-33600 Pessac- Bordeaux, France

[4] Department of Biomedical Engineering, The George Washington University, Washington, DC, 20052 USA

Supplementary Methods

S1 Section. Cell model equations and parameters.

The three cell types consisting of sinoatrial node (SAN), atrial cell, and paranodal cell type were simulated using a modified Fenton-Karma model [1]. The equations used were as follows:

$$\begin{aligned}
\partial u / \partial t &= \nabla D(x,y,z)\nabla u - J_{fi} - J_{so} - J_{si} \\
\partial v / \partial t &= \Theta(u_c - u)(1-v)\tau_v^{-} - \Theta(u-u_c)v\tau_v^{+} \\
\partial w / \partial t &= \Theta(u_c - u)(1-w)\tau_w^{-} - \Theta(u-u_c)w\tau_w^{+} \\
J_{fi} &= -v\Theta(u_c - u)(1-u)(u_c - u)/\tau_d \\
J_{so} &= u\Theta(u_c - u)/\tau_o + \Theta(u-u_c)/\tau_r \\
J_{si} &= -w(1+\tanh[k(u-u_c^{si})])/\tau_{si}
\end{aligned} \qquad \text{Equation S1}$$

No-flux boundary conditions were implemented:

$$\vec{n}.D(x,y,z)\nabla u = 0 \ \text{ at } \ \partial\theta \qquad \text{Equation S2}$$

The boundary conditions were implemented by using the method described in Fenton et al. [2]. Model parameter values for the three cell types were adapted from a previous study [3] and are given in Table S1.

References

1. Fenton F, Karma A. Vortex dynamics in three-dimensional continuous myocardium with fiber rotation: Filament instability and fibrillation. Chaos. 1998;8(1):20-47. Epub 2003/06/05. doi: 10.1063/1.166311. PubMed PMID: 12779708.
2. Fenton FH, Cherry EM, Karma A, Rappel WJ. Modeling wave propagation in realistic heart geometries using the phase-field method. Chaos. 2005;15(1):13502. Epub 2005/04/20. doi: 10.1063/1.1840311. PubMed PMID: 15836267.
3. Podziemski P, Zebrowski JJ. A simple model of the right atrium of the human heart with the sinoatrial and atrioventricular nodes included. Journal of clinical monitoring and computing. 2013;27(4):481-98. Epub 2013/02/23. doi: 10.1007/s10877-013-9429-6. PubMed PMID: 23430363; PubMed Central PMCID: PMC3689917.
