# Supplementary material for: Computational assessment of the functional role of sinoatrial node exit pathways in the human heart
Source: PLoS One. 2017 Sep 5;12(9):e0183727. doi: 10.1371/journal.pone.0183727 (PMC5584965; doi:10.1371/journal.pone.0183727)
Supplement: S2 Section — (PDF) [file pone.0183727.s005.pdf]

Supplementary Data

Computational assessment of the functional role of sinoatrial node exit pathways in the human heart

Sanjay R Kharche[1*], Edward Vigmond[2, 3], Igor R Efimov[4], Halina Dobrzynski[1*]

[1] Institute of Cardiovascular Sciences, School of Medical Sciences, University of Manchester, Manchester, M13 9NT, UK

[2] University of Bordeaux, IMB, UMR 5251, F-33400 Talence, France

[3] IHU Liryc, Electrophysiology and Heart Modeling Institute, Fondation Bordeaux Université, F-33600 Pessac- Bordeaux, France

[4] Department of Biomedical Engineering, The George Washington University, Washington, DC, 20052 USA

Supplementary Methods

S2 Section. Objective method for filament tracking.

In several simulation experiments, the behaviour of scroll waves in the 3D model was used to elicit the function of the SEPs. The initiation of scroll waves at a specific location within the 3D model was implemented using the phase distribution method as described previously [1, 2]. The evolution of the scroll wave filament or analysis of consecutive voltage distributions were used to quantify the evolution of the scroll wave dynamics. To identify the filament, the phase singularity method was used [3] (Figure S1). The filament tracing algorithm relies on system specific estimates of certain parameters that are required to accurately estimate the location of the filaments. To permit an objective estimation, the method as illustrated in Figure S1 was implemented for each of our simulation results. In brief, voltage was recorded from a chosen location (Figure 3, A). The correlation between voltage at time t and voltage after a certain delay at time t+ τ was computed. The delay that gave a small correlation was used to generate a phase plot of (V(t), V(t+ τ) (Figure 3, B-C). The centroid of the phase plot (Figure 3, D) was computed using standard MATLAB functions. The centroid of the illustrated signal was found to be (0.5, 0.59) which was used to compute the phase of the voltage excitation, ϕ, at all locations within the 3D model (Figure 3, E):

$$\phi(t,\ x,\ y,\ z) = \tan^{-1}\left(\frac{V(t,\ x,\ y,\ z) \quad - \ 0.50}{V(t+tau,\ x,\ y,\ z) \ - \ 0.59} \right) \qquad \text{Equation S2}$$

The filament was defined as all the locations where the curl of the gradient of the phase was found to be multiples of ±2π as described in Bray et al. [3].

The number of filaments were counted using a generic grassfire algorithm. The filament coordinates were not manipulated for smoothness and extrapolated connectivity since that may lead to further introduction of errors.

References

1. Biktashev VN, Holden AV. Reentrant waves and their elimination in a model of mammalian ventricular tissue. Chaos. 1998;8(1):48-56. doi: Doi 10.1063/1.166307. PubMed PMID: ISI:000072635100003.

2. Kharche SR, Biktasheva IV, Seemann G, Zhang HG, Biktashev VN. A Computer Simulation Study of Anatomy Induced Drift of Spiral Waves in the Human Atrium. Biomed Res Int 2015; 2015: 731386 Epub 2015/10/26. 2015. doi: Artn 731386

10.1155/2015/731386. PubMed PMID: ISI:000364683800001.

3. Bray MA, Wikswo JP. Use of topological charge to determine filament location and dynamics in a numerical model of scroll wave activity. IEEE Trans Biomed Eng. 2002;49(10):1086-93. Epub 2002/10/11. doi: 10.1109/TBME.2002.803516. PubMed PMID: 12374332.
