# Supplementary material for: Computational assessment of the functional role of sinoatrial node exit pathways in the human heart
Source: PLoS One. 2017 Sep 5;12(9):e0183727. doi: 10.1371/journal.pone.0183727 (PMC5584965; doi:10.1371/journal.pone.0183727)
Supplement: S1 Table — Control values are given in black, and ISO (short SAN AP, short atrial AP) as well as Ach values (long SAN AP, short atrial AP) are given in red. (PDF) [file pone.0183727.s006.pdf]

Supplementary Data

Computational assessment of the functional role of sinoatrial node exit pathways in the human heart

Sanjay R Kharche[1*], Edward Vigmond[2, 3], Igor R Efimov[4], Halina Dobrzynski[1*]

[1] Institute of Cardiovascular Sciences, School of Medical Sciences, University of Manchester, Manchester, M13 9NT, UK

[2] University of Bordeaux, IMB, UMR 5251, F-33400 Talence, France

[3] IHU Liryc, Electrophysiology and Heart Modeling Institute, Fondation Bordeaux Université, F-33600 Pessac- Bordeaux, France

[4] Department of Biomedical Engineering, The George Washington University, Washington, DC, 20052 USA

Supplementary Methods

S1 Table. Model parameter values in the cell types of human SAN model. Control values are given in black, and ISO (short SAN AP, short atrial AP) as well as Ach values (long SAN AP, short atrial AP) are given in red.

Parameter	SAN	Paranodal	atrial
u_c	0.1	0.1	0.3
u_v	0.5	0.5	0.5
τ_d	0.05	0.05	0.2
τ_0	12.5	12.5	12.5
τ_{si}	50	50	127
τ_r	130	130	130
τ_v^+	10	10	10
τ_v^-	18.2	18.2	18.2 (short AP due to ISO and Ach: 30)
τ_w^+	1.5	1.5	1020 (short AP due to ISO and Ach: 10)
τ_w^-	850 (ISO induced fast pacing using 400. Ach induced slow pacing using 1200)	1400	80 (short atrial AP due to ISO and Ach: 352)
u_c^{si}	0.01	0.01	0.85
k	5	5	10

30
